# Reconstructing neuronal circuitry from parallel spike trains

**DOI:** 10.1038/s41467-019-12225-2

**Published:** 2019-10-02

**Authors:** Ryota Kobayashi, Shuhei Kurita, Anno Kurth, Katsunori Kitano, Kenji Mizuseki, Markus Diesmann, Barry J. Richmond, Shigeru Shinomoto

**Affiliations:** 1 National Institute of Informatics, Tokyo, 101-8430 Japan; 2 Department of Informatics, SOKENDAI (The Graduate University for Advanced Studies), Tokyo, 101-8430 Japan; 3 Center for Advanced Intelligence Project, RIKEN, Tokyo, 103-0027 Japan; 4 Institute of Neuroscience and Medicine (INM-6) and Institute for Advanced Simulation (IAS-6) and JARA-Institute Brain Structure-Function Relationships (INM-10), Jülich Research Centre, 52425 Jülich, Germany; 5 Department of Physics, Faculty 1, RWTH Aachen University, Aachen, Germany; 6 Department of Information Science and Engineering, Ritsumeikan University, Kusatsu, 525-8577 Japan; 7 Department of Physiology, Osaka City University Graduate School of Medicine, Osaka, 545-8585 Japan; 8 Department of Psychiatry, Psychotherapy and Psychosomatics, School of Medicine, RWTH Aachen University, Aachen, Germany; 9 Laboratory of Neuropsychology, NIMH/NIH/DHHS, Bethesda, MD 20814 USA; 10 Department of Physics, Kyoto University, Kyoto, 606-8502 Japan; 11 Brain Information Communication Research Laboratory Group, ATR Institute International, Kyoto, 619-0288 Japan

**Keywords:** Neuroscience, Computational neuroscience, Neural decoding

## Abstract

State-of-the-art techniques allow researchers to record large numbers of spike trains in parallel for many hours. With enough such data, we should be able to infer the connectivity among neurons. Here we develop a method for reconstructing neuronal circuitry by applying a generalized linear model (GLM) to spike cross-correlations. Our method estimates connections between neurons in units of postsynaptic potentials and the amount of spike recordings needed to verify connections. The performance of inference is optimized by counting the estimation errors using synthetic data. This method is superior to other established methods in correctly estimating connectivity. By applying our method to rat hippocampal data, we show that the types of estimated connections match the results inferred from other physiological cues. Thus our method provides the means to build a circuit diagram from recorded spike trains, thereby providing a basis for elucidating the differences in information processing in different brain regions.

## Abstract (web summary)

Current techniques have enabled the simultaneous collection of spike train data from large numbers of neurons. Here, the authors report a method to infer the underlying neural circuit connectivity diagram based on a generalized linear model applied to spike cross-correlations between neurons.

## Introduction

Over the past decade it has become possible to record from much larger numbers of neurons than in the past^1–5^, even though this number is still a mere shadow of the total number of neurons present. The premise behind collecting these large data sets is that this could lead to improvements in correlating neuronal activity with specific sensations, motion, or memory, and possibly lead to improvements in adaptation and learning as well^6–10^.

Having such large data sets leads to difficulties in handling the data and interpreting the results. There are two main approaches to handle large amounts of recording data. In the first approach, researchers have developed methods to reduce dimensionality while minimizing the loss of information^11–13^.

The second approach, which we take here, is to use all of the data to carry out mesoscopic neuroanatomy, that is, to reveal the fine neuronal circuitry in which neural circuit computation is carried out. From these high channel count recordings, one should be able to estimate neuronal connectivity by quantifying the degree to which firing from a given neuron is influenced by the firing of neurons from which the index neuron is receiving input^14–30^. For this purpose, we develop an analytical tool that estimates neuronal connectivity in measurement units of postsynaptic potentials (PSPs). In this study we also investigate how much data are needed to reliably estimate the connections between pairs of neurons. Because reconstructing connectivity is not guaranteed to reflect anatomical connectivity^31–33^, we evaluate the accuracy of estimation by directly comparing the estimated connections with the true connections, using synthetic data generated by simulating a network of Hodgkin–Huxley (HH)-type neurons or a large network of leaky integrate-and-fire (LIF) neurons. Finally, we apply this method to spike trains recorded from rat hippocampus. For the experimental data, we compare our estimates of whether an innervating connection is excitatory or inhibitory with the results obtained by manually analyzing other physiological information such as spike waveforms, autocorrelograms, and mean firing rate.

## Results

### Estimating neuronal connections

**Fig. 1:**
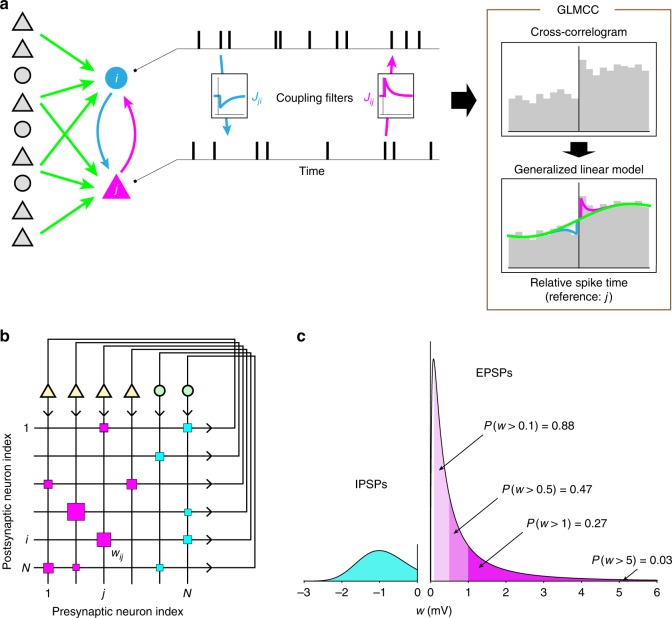
Estimating neuronal connections. **a** Connectivity between neurons is estimated by fitting a generalized linear model (GLM) to the cross-correlation (CC). ${J}_{ij}$ represents a coupling from the $j$-th neuron to the $i$-th neuron. Excitatory and inhibitory neurons are depicted as triangles and circles, and their synaptic connections are colored magenta and cyan, respectively. Surrounding neurons may induce large-scale fluctuations in the CC (light green line). **b** Neuronal connectivity is visualized by the Hinton diagram, in which excitatory and inhibitory connections are represented, respectively by magenta and cyan squares of the sizes (area) proportional to the postsynaptic potential (PSP) ${w}_{ij}$. **c** Distributions of excitatory postsynaptic potentials (EPSPs) and inhibitory postsynaptic potentials (IPSPs) of a simulated network

To estimate neuronal connectivity between each pair of neurons, we obtain the cross-correlation (CC) by collecting spike times of a neuron measured relative to every spike of a reference neuron (Fig. 1a). We explore the CC for the evidence of a monosynaptic impact of a few milliseconds using the generalized linear model (GLM). Here, neuronal connectivity is detected by fitting a coupling filter, while slow, large-scale wavy fluctuations that are often present in recorded spike trains are absorbed by adapting the slow part of the GLM. We call our method “GLMCC” (METHODS).

### Criterion for the presence of connections

A neuronal connection is considered significant when the estimated parameter falls outside the confidence interval of a given significance level for the null hypothesis that the connection is absent. If the parameter remains within the confidence interval, the state of the connection is undetermined (METHODS).

The number of pairs considered to be connected will depend on the significance level $\alpha$ and on the strength of the correlation. Estimation methods presume connections as if they were all direct ones, causing strong indirect influences to be purported direct connections. Neurophysiologists often try to avoid these false positives (FPs) by shifting the significance level to small values, that is, by moving $\alpha$ to very stringent levels. However, being conservative about FPs means that existing connections important for information processing will be missed, thereby producing many false negatives (FNs).

To capture the manner in which the numbers of FPs and FNs change with the level of conservatism used for estimating connections, we applied our inference model to spike trains obtained from a network of HH neurons, in which the true anatomical connectivity is known. With this knowledge, we searched for the optimal level of conservatism or the significance level that may balance the conflicting demands for reducing FPs and FNs.

Our simulation used a network of 1000 HH neurons consisting of 800 excitatory and 200 inhibitory neurons (cf. Fig. 1b). In the simulation, excitatory neurons innervated 12.5% of other neurons with excitatory postsynaptic potentials (EPSPs). These excitatory connections were log normally distributed^34–37^ (Fig. 1c). Inhibitory neurons randomly innervated 25% of other neurons with inhibitory postsynaptic potentials (IPSPs). These inhibitory connections were normally (Gaussian) distributed^38^. We simulated the network for a period representing 5400 s (90 min) with step sizes of 0.01 and 0.001 ms for excitatory and inhibitory neurons, respectively (METHODS). Our simulation reproduced irregular neuronal firing and skewed distribution of firing rates, which are consistent with balanced state network models^39^ (Supplementary Fig. 1).

**Fig. 2:**
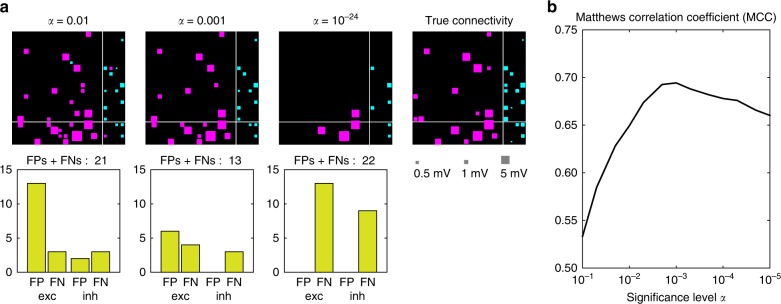
Selecting the significance level. **a** The connection matrices are estimated with different levels of conservatism against making false positives (FPs), which are represented by the significance level $\alpha$. In each connection matrix, the $x$-axis indicates reference (index) neurons. The connection matrix is divided into four quadrants representing inhibitory–excitatory, excitatory–excitatory, excitatory–inhibitory, and inhibitory–inhibitory zones. The numbers of FP and false negative (FN) connections for the excitatory and inhibitory categories are depicted below the matrices. In the true connectivity, weak excitatory connections whose EPSPs are smaller than 1 mV are not considered. **b** The Matthews correlation coefficient (MCC) is plotted against the significance level $\alpha$. The MCC takes a maximum at an intermediate level of cautiousness, given by $\alpha\, =\,0.001$. Source data are provided as a Source Data file

To illustrate the performance of estimating connections, we sampled 20 neurons out of the entire population. Figure 2a shows the estimated connection matrices obtained using different significance levels, in reference to the true connectivity. Here we have not considered weak excitatory connections whose EPSPs are smaller than 1 mV, because the amount of spike recording is insufficient for identifying connections of this level. The connection matrix is divided into four quadrants representing connections between inhibitory–excitatory, excitatory–excitatory, excitatory–inhibitory, and inhibitory–inhibitory neurons. True connections for the second and third quadrants are excitatory, and those of the fourth and first quadrants are inhibitory. For $\alpha =0.01$, too many false connections were assigned to pairs of neurons; there were 15 false connections (4.3%) in this sample. At the other extreme, all FPs can be excluded by decreasing the significance level (down to $\alpha =1{0}^{-24}$). In the latter case most existing connections are lost, and a large number of FNs arise; 22 among 32 existing connections (69%) are missed in this example. The numbers of FPs and FNs for excitatory and inhibitory categories are shown below for the connection matrices, indicating that the total number of FPs and FNs may be minimized between these extreme cases.

To balance the FPs and FNs simultaneously, we selected the significance level that maximized the Matthews correlation coefficient (MCC)^27,^^40^. The significance level was set to $\alpha\, =\,0.001$ (Fig. 2b). Although false connections remain, the neuronal circuit was most accurately reconstructed with $\alpha \,=\,0.001$. We adopted $\alpha\, =\,0.001$ throughout the following analyses.

### Duration of spike recording

The necessary duration of spike recording can be estimated even without fitting the statistical model to the spike trains. This is because the distribution of the connection parameter for the null hypothesis is obtained solely in terms of the observation interval ($T$) and the firing rates of the pre and postsynaptic neurons (${\lambda }_{{\rm{pre}}}$ and ${\lambda }_{{\rm{post}}}$) (METHODS). The confidence interval of the connection parameter ($J$) is

1 $${J}_{\pm }\,=\,\pm c/{\left(T\tau {\lambda }_{{\rm{pre}}}{\lambda }_{{\rm{post}}}\right)}^{1/2},$$

where $\tau$ is the time scale of synaptic impact, which is chosen by maximizing the model likelihood: $\tau\, =\,0.004$ s for the simulation data and $\tau \,=\,0.001$ s for the rat hippocampal data. The coefficient $c$ is given as 5.16 for $\alpha \,=\,0.001$.

We assume that connection parameter $J$ is proportional to the PSP, $w$ mV^41^:

2 $$J\,=\,aw.$$

The coefficient $a$ is determined using synthetic data as $a\,=\,0.39$ for the EPSP and $a\,=\,1.57$ for IPSP. By combining this with Eq. (1), the necessary duration of spike recording needed to determine the likely presence of a connection of PSP is given as

3 $$T\,> \,\frac{{c}^{2}}{\tau {\lambda }_{{\rm{pre}}}{\lambda }_{{\rm{post}}}{a}^{2}{w}^{2}}.$$

According to the coefficient $a$, which is larger for IPSP than for EPSP, the inhibitory connection is detected more easily than the excitatory connection, given the same PSP $|w|$. This is in conflict with the results of some other studies^16,42,43^. The disagreement is due to the difference in simulation models; in our simulation model, the time scale of the inhibitory synapse is chosen to be longer than that of the excitatory synapse on the basis of physiological experiments^44,45^. Accordingly, the inhibitory response is slower and has a larger integrated effect than the excitatory response. Our GLMCC should be able to properly detect the overall integrated effect (Supplementary Fig. 2).

To make reliable inference, in addition to the above relation, it is also necessary to have collected a sufficiently large number of spikes during the interaction time window on the order of a few milliseconds. Here we require (METHODS):

4 $$T{\lambda }_{{\rm{pre}}}{\lambda }_{{\rm{post}}}\,> \,10/\tau \, [{{\rm{s}}}^{-1}].$$

**Fig. 3:**
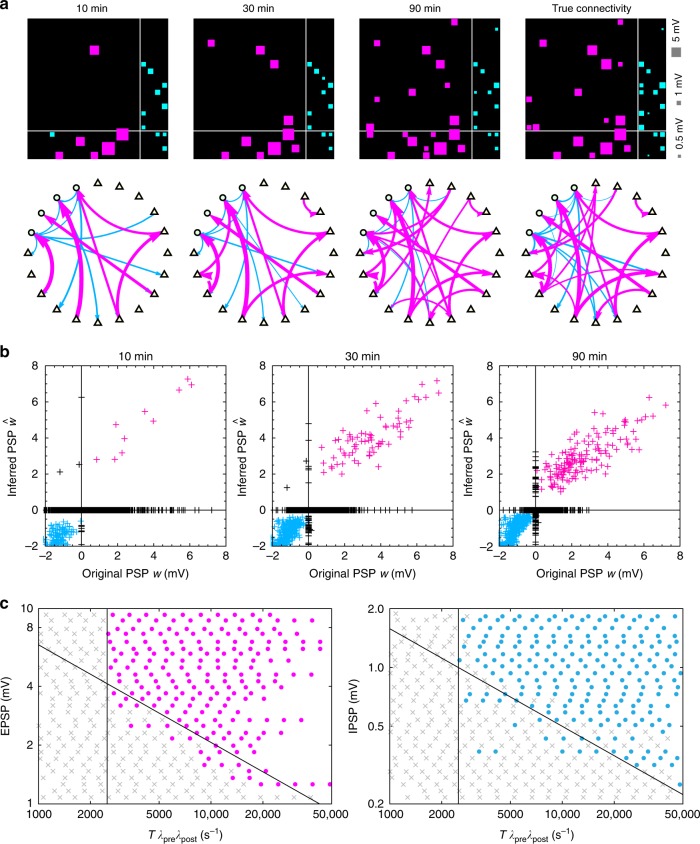
Neuronal circuits reconstructed from different observation time windows. **a** Neuronal connections estimated from the observation time windows of 600, 1800, and 5400 s (10, 30, and 90 min) are plotted in reference to true connectivity. In each connection matrix, the $x$-axis indicates reference neurons. In the network graphs shown in the second panel, excitatory and inhibitory neurons are depicted as triangles and circles, respectively. **b** Estimated postsynaptic potentials (PSPs) ($\hat{w}$) plotted against true parameters ($w$) were computed for 100 neurons randomly selected from the simulation. Points in the first and third quadrants represent qualitatively correct inferences for excitatory and inhibitory connections (magenta and cyan, respectively). Points on the nonzero $y$-axis represent the false positive connections for unconnected pairs. Points on the nonzero $x$-axis represent the false negatives. **c** Detection status for connections of given PSPs with respect to the observation window ($T$). Connections estimated as excitatory and inhibitory are colored magenta and cyan, respectively, while undetermined ones are colored gray. Diagonal and vertical lines represent the theoretical formulas (3) and (4), respectively. Source data are provided as a Source Data file

Table 1 shows the results of several cases of firing rates and the assumed PSPs using the $\alpha =0.001$. Unsurprisingly, to detect a weak connection for a low firing neuron requires gathering data for a long period of time. Figure 3a shows the connections estimated with different observation time windows, illustrating how weak connections become visible as the recording duration increases.

**Table 1:** Duration of spike recording required for verifying neuronal connections

Firing rates	EPSP: 5 mV	EPSP: 1 mV	EPSP: 0.5 mV	IPSP: 1 mV	IPSP: 0.5 mV
(10, 10) Hz	$T\,> \,2$ min	$T\,> \,30$ min	$T\,> \,2$ h	$T\,> \,2$ min	$T\,> \,7$ min
(10, 5) Hz	$T\,> \,3$ min	$T\,> \,1$ h	$T\,> \,4$ h	$T\,> \,4$ min	$T\,> \,10$ min
(5, 5) Hz	$T\,> \,7$ min	$T\,> \,2$ h	$T\,> \,8$ h	$T\,> \,7$ min	$T\,> \,30$ min
(10, 1) Hz	$T\,> \,20$ min	$T\,> \,5$ h	$T\,> \,20$ h	$T\,> \,20$ min	$T\,> \,1$ h
(5, 1) Hz	$T\,> \,30$ min	$T\,> \,10$ h	$T\,> \,40$ h	$T\,> \,40$ min	$T\,> \,2$ h
(1, 1) Hz	$T\,> \,3$ h	$T\,> \,50$ h	$T\,> \,200$ h	$T\,> \,3$ h	$T\,> \,10$ h

In the first column, the two numbers in the bracket represent the firing rates of pre and postsynaptic neurons, or post and presynaptic neurons. This table is obtained with the synaptic time scale τ = 1 ms, which was selected for rat hippocampal data

### Estimating PSPs

We believe that our method is of particular interest because it couches the connections in terms of PSPs for the individual neuronal pairs. Figure 3b compares the estimated PSPs ($\hat{w}$) against the true values ($w$) from the numerical simulation. Here we represent $\hat{w}\,=\,0$ if the connection is undetermined, i.e., not significant. Thus, unconnected links ($w\,=\,0$) that were classified as undetermined (true negatives) are placed at the origin. Points lying on the nonzero $x$-axis are existing connections that were not detected. Points lying on the nonzero $y$-axis are the functional or virtual connections that were estimated for unconnected pairs. The points in the first and third quadrants represent true positives, or existing connections whose signs were correctly inferred as excitatory or inhibitory, respectively. The points in the second and fourth quadrants are existing connections whose signs were misclassified.

The number of nonzero connections increases with the recording duration. Existing connections with large PSP amplitude tend to be detected with the signs correctly identified (points in the first and third quadrants). There are also virtual connections assigned for unconnected pairs (nonzero $y$-axis). The number of such FPs is larger than the expected number of statistical errors (Fig. 2a). This implies that the false connections may not be mere statistical fluctuations, but rather that they may reflect the functional connectivity indirectly connected via other unobserved neurons.

Figure 3c demonstrates the way individual connections emerge by increasing the recording duration. Here the abscissa is the observation window ($T$) multiplied by the firing rates of the pre and postsynaptic neurons (${\lambda }_{{\rm{pre}}}$ and ${\lambda }_{{\rm{post}}}$) so that all data are organized into a unified formula (inequality (3)). The values of $T{\lambda }_{{\rm{pre}}}{\lambda }_{{\rm{post}}}$ for the excitatory connections tended to be smaller than those of inhibitory connections, because the firing rates of excitatory neurons were typically lower than those of inhibitory neurons.

### Excitatory–inhibitory (E–I) dominance index

The probability of misassigning individual connectivity for unconnected pairs tends to be higher than the statistical significance level, because their firing is generally correlated with each other due to indirect interactions through unobserved neurons. Nevertheless, excitatory and inhibitory characteristics of individual neurons can be inferred with a lower error rate, because we can refer to multiple connections for each neuron.

**Fig. 4:**
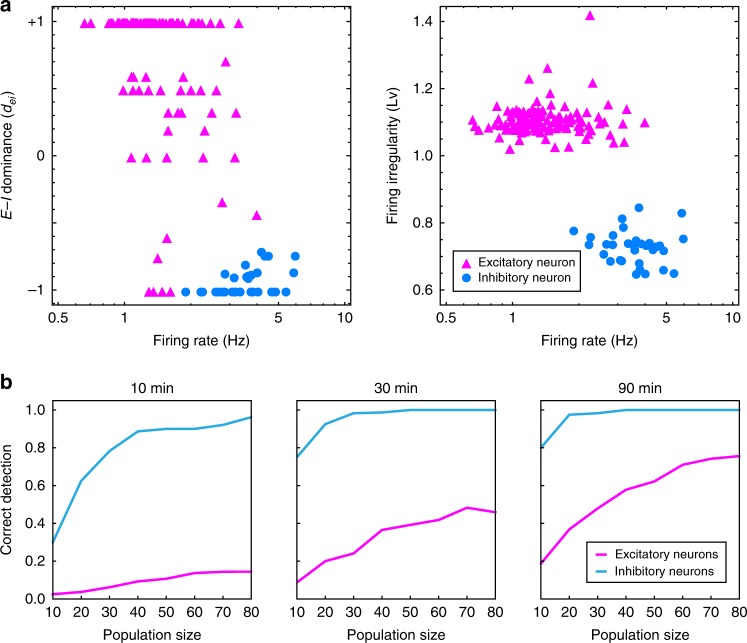
Excitatory–inhibitory (E–I) dominance index. **a** E–I dominance index ${d}_{{\rm{ei}}}\,=\,({n}_{{\rm{e}}}\,-\,{n}_{{\rm{i}}})/({n}_{{\rm{e}}}\,+\,{n}_{{\rm{i}}})$ and the firing irregularity ($Lv$), plotted against the firing rate for 160 HH-type neurons (2 networks of 80 neurons). Excitatory and inhibitory neurons are plotted as triangles and disks, colored magenta and cyan, respectively. **b** The rates at which excitatory and inhibitory characteristics are identified correctly according to ${d}_{{\rm{ei}}}\,> \,0$ and ${d}_{{\rm{ei}}}\,<\,0$, respectively for excitatory and inhibitory neurons. Source data are provided as a Source Data file

We define an excitatory–inhibitory (E–I) dominance index as

5 $${d}_{{\rm{ei}}}\,=\,\frac{{n}_{{\rm{e}}}\,-\,{n}_{{\rm{i}}}}{{n}_{{\rm{e}}}\,+\,{n}_{{\rm{i}}}}.$$

where ${n}_{{\rm{e}}}$ and ${n}_{{\rm{i}}}$ represent the numbers of identified excitatory and inhibitory connections projecting from each neuron, respectively. The E–I dominance indexes computed for 2 networks of 80 neurons each are plotted against firing rates of neurons (Fig. 4a). In this case, excitatory and inhibitory characteristics of individual neurons were well-identified based on E–I dominance indexes. Inhibitory neurons typically exhibited higher firing rates in comparison to excitatory neurons. The firing irregularity measured using the local variation ($Lv$) of interspike intervals^46,47^ is plotted against firing rate. Spiking of inhibitory neurons tended to be more regular (smaller $Lv$) than that of excitatory neurons.

If we can record many spike trains in parallel for a long time, many excitatory and inhibitory neurons may be correctly identified according to ${d}_{{\rm{ei}}}\,> \,0$ and ${d}_{{\rm{ei}}}\,<\,0$, respectively. Figure 4b illustrates the manner in which the ratio of such correct identification depends on the total number of spike trains and the duration of observation.

### Real spike trains

**Fig. 5:**
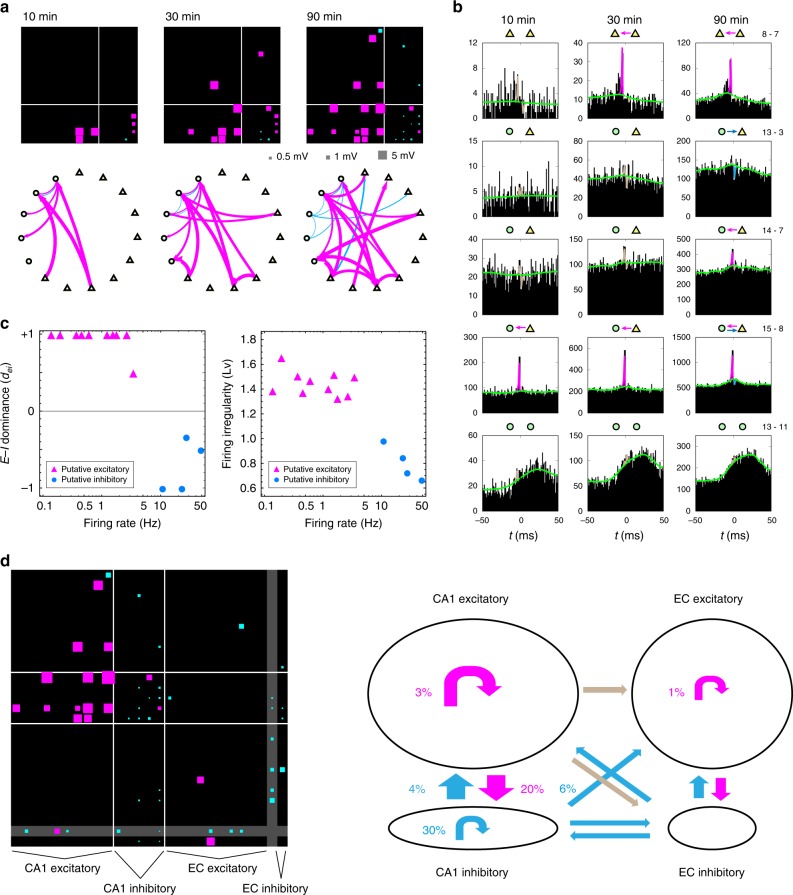
Neuronal circuits reconstructed from real spike trains in vivo. **a** Neuronal connections estimated from spike trains recorded from the hippocampal CA1 area of a rat. Estimations were made with observation time windows of 600, 1800, and 5400 s (10, 30, and 90 min) for neurons whose firing rate is $> 0.5$ [Hz]. In each connection matrix, the $x$-axis indicates reference neurons. The connection matrix is partitioned into groups of putative excitatory and inhibitory neurons defined manually according to other physiological cues such as waveforms. **b** Cross-correlations of several pairs of neurons computed at different time windows. The slow part of the GLM adapted to the data is depicted as a light green line. The coupling filter is separately depicted in magenta, cyan, or gray, for the excitatory, inhibitory, or undetermined, respectively. Corroborated connections are indicated by arrows. **c** E–I dominance index (${d}_{{\rm{ei}}}$) and firing irregularity ($Lv$) are plotted against the firing rates for putative excitatory and inhibitory neurons. Neurons with $> 1$ connection with firing rate and $> 0.1$ [Hz] are plotted in the E–I dominance index. **d** Estimated connections among neurons in CA1 and Entorhinal Cortex (EC). The connection matrix is partitioned into putative excitatory and inhibitory neurons in CA1 and EC. One EC unit, whose excitatory or inhibitory characteristic was not determined by the manual analysis, is put in the gap (gray) between excitatory and inhibitory groups. In the network graph shown in the second panel, excitatory- and inhibitory-dominated connections are depicted in magenta and cyan, while connections of mixed characteristics are depicted in gray. Source data are provided as a Source Data file

We apply our method to spike trains recorded from the hippocampal CA1 area of a rat while it was exploring a square open field (hc-3 data sets in Collaborative Research in Computational Neuroscience (CRCNS))^48^. Figure 5a displays the connections obtained with different observation time windows, demonstrating that more connections become visible as the recording duration increases, similar to the results seen with synthetic data. The connection matrix is divided into four quadrants according to the putative classification performed by manually analyzing waveforms, autocorrelograms, and mean firing rates^49–51^. We observe that connections in the third, fourth, and first quadrants of the connectivity matrix representing excitatory–inhibitory, and inhibitory–inhibitory, and inhibitory–excitatory zones, respectively, are detected in a relatively short observation window. This is consistent with our formula (3), given that inhibitory neurons typically fire at high rates, though inhibitory neurons are not necessarily a uniform population^52^. Connections in the second quadrant, representing the excitatory–excitatory zone, only appear after increasing the observation time window, and the estimated connection pattern remains sparse; more connections might have been identified if the observation period had been even longer. However, the estimated connection pattern is consistent with the finding using intracellular recording in vitro that inter-pyramidal connections in the hippocampus CA1 are sparse^53^.

Figure 5b shows CCs of several neuron pairs (see Supplementary Fig. 3 for all the detected pairs). Here, we have excluded spike records at an interval of $\pm 1$ ms in the cross-correlogram, because near-synchronous spikes were not detected in the experiment due to the shadowing effect^54^. The CCs become less noisy as the observation time increases, and some connections resolved (8–7, 13–3, 14–7, and 15–8). Some real spike trains exhibited large-scale wavy fluctuations (13–11), which may suggest that these neurons are under the influence of brain activity with lagged phases or perhaps they were responding to some unidentified external stimulus. Our method absorbs these fluctuations by adapting the slow part of the GLM (demonstrated as light green lines in Fig. 5b), and succeeds in detecting a tiny impact by fitting coupling filters (lines colored magenta, cyan, and gray, respectively represent excitatory, inhibitory, and undetermined connections in Fig. 5b).

In Fig. 5c, we plotted the E–I dominance index (${d}_{{\rm{ei}}}$) and the firing irregularity ($Lv$) against the firing rate. The E–I dominance index is roughly consistent with the putative excitatory and inhibitory neurons. The irregularity of the putative excitatory neurons tended to be higher (larger $Lv$) than that of the inhibitory neurons, similar to what we observed with the simulation data. The good separation of the putative excitatory and inhibitory neurons in these plots implies that we can classify recorded cells into excitatory and inhibitory neurons reliably without having to rely on their waveforms, as the E–I dominance index, firing irregularity, and firing rate are obtained solely from the spike times.

We also attempted to analyze a set of spike trains recorded simultaneously from multiple regions including CA1 and the Entorhinal Cortex (EC). Figure 5d demonstrates a matrix of estimated connections among excitatory and inhibitory neurons in CA1 and EC. Though the number of inter-regional connections was small in this sample data, our analysis method is generally applicable to any set of spike trains, irrespective of the recorded areas.

### Comparison with other methods

**Fig. 6:**
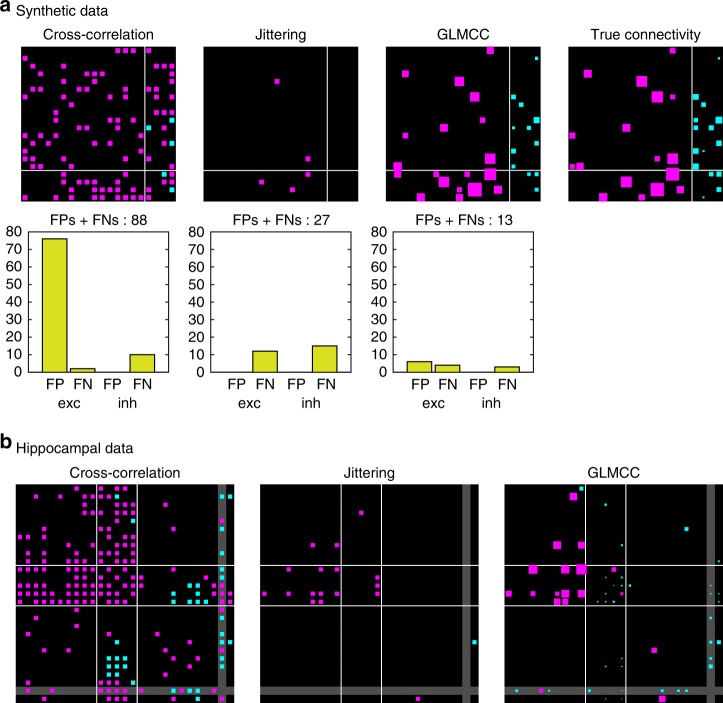
Comparison of estimation methods. **a** Connections estimated using the conventional cross-correlation method, the jittering method, and our GLMCC method, in reference to the true connectivity of the synthetic data (used in Fig. 3). For the GLMCC and the true connectivity, the size of each square is proportional to the PSP amplitude, while for the first two methods, the estimated connections are represented in equal size, because they do not estimate the PSP. **b** Neuronal connections estimated from spike trains recorded from the hippocampus of a rat (used in Fig. 5d). Source data are provided as a Source Data file

We compared our method with the conventional CC method^16^ and the jittering method^25^ by applying these methods to synthetic and biological data. With the synthetic data, we can compare the performance of inferring connectivity with the true connectivity (Fig. 6a). Here, we have not shown excitatory connections smaller than 1 mV in the true connectivity matrix as in Fig. 2a, because they are unlikely to be detected in a 90 min recording. The relative performance of the analysis methods is unchanged even if the smaller EPSPs are included. The conventional CC analysis tended to produce a number of FPs, revealing a vulnerability to fluctuations in cross-correlograms. In contrast, the jittering method avoided making FPs, but missed many existing connections, in particular for inhibitory connections. This result may have occurred because the decrease in the firing rate induced by an inhibitory interaction is slower than an impulsive response to an excitatory stimulus; the jittering method count spikes in each bin and tends to overlook a slower modulation in the firing rate. The number of false connections was 88, 27, and 13, respectively for the conventional CC method, the jittering method, and the GLMCC method, indicating the superiority of the present method. We also examined the manner in which the number of errors varies with the firing rate of neurons, and found that the estimation error increases with the firing rates (Supplementary Fig. 4).

We also compared the connections estimated from the real biological data recorded from the hippocampus of a rat (Fig. 6b). The conventional CC method and jittering method suggested many (false) excitatory connections from putative inhibitory neurons to other neurons. In the GLMCC, most of detected inhibitory connections in hippocampal data are from inhibitory to inhibitory or from inhibitory to excitatory neurons, consistent with low FPs and FNs in inhibitory connections in synthetic data by this method.

### Testing with large-scale simulations

We have tuned the GLMCC method using synthetic data of a network of 1000 HH neurons and assessed the estimation performance. We have also tested the method with simulation data of different inhibitory connectivities and those generated by LIF neurons^29^, and confirmed that the method estimates the connectivity accurately for these data as well (Supplementary Fig. 5). In the original simulation, 1000 HH neurons are densely connected with excitatory neurons innervating EPSPs to 12.5% of other neurons. However, the effective connectivity is rather sparse, because the EPSPs are log normally distributed and the majority of them are weak. Accordingly, the number of effective connections each neuron receives is not large in this network size.

**Fig. 7:**
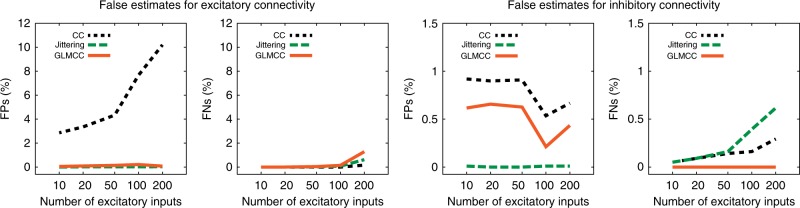
The number of estimation errors computed for networks of 10,000 LIF neurons. Horizontal axes represent the average number of excitatory inputs to each neuron. For excitatory (inhibitory) connectivity, FPs represent directed links that were mistakenly assigned as excitatory (inhibitory), whereas FNs represent excitatory (inhibitory) connections that were assigned as disconnected or inhibitory (excitatory). Source data are provided as a Source Data file

Considering the realistic situation in which each neuron is receiving strong connections from a number of neurons, we carried out simulations of a larger scale network consisting of 10,000 LIF neurons using the NEST simulator^55^ (Supplementary Note 1 and Supplementary Tables 1, 2, and 3). By performing simulations of different connection densities, we examined the manner in which the number of false estimates varies with the number of connections. Figure 7 demonstrates the proportions of FPs and FNs counted for each pair of neurons, indicating the stable estimation of the GLMCC method and its superiority to other existing methods. Sample connectivity matrices are presented in Supplementary Fig. 6.

## Discussion

We have presented a method for reconstructing neuronal circuitry from multichannel extracellular neuronal recordings. This method, based on a combination of the GLM and CC, can balance the antagonistic demands for reducing FPs and FNs when estimating neuronal connectivity. Our method is tolerant of the large variations in firing activity that often occur in vivo. As a critical part of the method, we show a framework for estimating the necessary duration of the spike recordings so that any likely neuronal connections are detected. The duration is presented in terms of the firing rates of the pre- and postsynaptic neurons, and the presumed PSP.

It would be ideal to be able to estimate individual connections using intracellular or patch clamp recordings where the postsynaptic current caused by presynaptic neuronal firing can be measured, as is done with recordings from the rat cortex^34,37^. While those methods can reliably detect synaptic connections, they are limited because only a few neurons can be recorded simultaneously.

With the recent increase in parallel high channel count extracellular recordings from anaesthetized and behaving animal subjects^1,2^, it is possible to estimate the connection strength between a number of neurons^20,28^. Several strong analytical methods for estimating connections from spike trains have been developed, including the CC analysis^14,15,18,21^ and the GLM^8,19,23,27,29^. While CCs have been used to estimate neuronal connectivity, this classical CC analysis becomes unreliable when there are large fluctuations in the data. One approach to solving this problem has been to jitter the time stamps of spikes^25,28^. We tested the performance of the conventional CC method and the jittering method in estimating connectivity using synthetic data, and found that our GLMCC performed better than conventional methods (Fig. 6).

Another approach has been to apply GLM to parallel spike trains. However, the size of the computation increases as the recording time increases. Because the number of neuronal pairs increases by the square of the number of spike trains (e.g., 10,000 pairs should be examined for 100 parallel spike trains), computation for estimating individual connections of each pair should be modest. Our analysis can be conducted with a reasonable computation time with amounts of data that can reasonably be collected, as our GLM analyses the CC for a time window of 100 ms rather than the entire spike trains. Our GLMCC may also adapt to wavy fluctuations in CC, making it tolerant to large-scale fluctuations that are often attendant on real spike trains in vivo (cf. Fig. 5b). There could also be fluctuations on an even longer time scale. There are several methods for processing such nonstationarity, including the state-space models^56,57^ or the Gaussian process^58^. Such slow fluctuations may induce variation in the CC amplitude, but they would not appear in the averaged cross-correlogram $c(t)$ in an interval of 100 ms in our framework.

In general, biological data are accompanied with large nonstationary fluctuations; neuronal firing rate may change according to behavioral contexts, and it might even occur that each neuron may appear or disappear due to unstable recording. To examine whether our method may have provided consistent estimation for neuronal connections, we split the recordings in half and compared estimated connections from each half (Supplementary Fig. 7). We found that the estimated connections exhibited significant overlap between the first and second halves. Thus, our inference method provides consistent data, not only for synthetic data, but also for experimental data. It is interesting to test our estimation with the information of biological connectivity, which is obtained by the latest experimental techniques such as the intracellular current injection^59^ or optogenetic control^60^.

Because recording time is limited, a possible restriction on inferring connectivity could be that there is not enough data. Here we made estimates on the duration of spike recordings needed so that any likely neuronal connections would be detected (cf. Table 1). It should be noted that the limit given in Eq. (3) or Table 1 is not due to a limitation of our method, but it is an essential limitation caused by the sparse firing itself. Even if a given neuron fired several times with each spike occurring shortly after the firing of an index neuron, such evidence may not be sufficient to confirm the presence of a synaptic connection. Thus, enough data are needed so that spike co-occurrence becomes statistically significant^28^.

When we applied our method to data recorded from the rat hippocampus we identified connections for four types of pairs including excitatory–excitatory, excitatory–inhibitory, inhibitory–inhibitory, and inhibitory–excitatory. These numbers were consistent with those identified physiologically^51^, supporting the efficacy of our method. Typically, the pyramidal neurons have low background firing rates and interneurons have higher firing rates. Our analysis (cf. inequality (3)) indicates that the necessary recording duration is inversely proportional to the product of the firing rates of the pre and postsynaptic neurons. Thus, connections between neurons firing at high frequencies can be detected with a relatively short observation duration. In contrast, for neurons with low firing rates, data will have to be collected for much longer periods, and we expect that excitatory–excitatory connections will be detected only if there is a relatively long recording period. The consequences of this have been seen with experimental data; for instance, synapses that connect with inhibitory interneurons were frequently detected, and connections between excitatory neurons were rarely detected^20^^,61^. The hippocampal data analyzed in this study (Fig. 5a) conforms to this pattern, and our analysis provides insight into how this happens. Our approach and method provide a means for estimating a map of neuronal connections from high channel count simultaneous recordings. We presume, based on anatomical differences, that these maps will have different structures in different functional brain regions. Having a reliable technique for estimating the maps offers the opportunity to identify these different structures, thereby providing a basis for understanding the variations in information processing that arises from differences in anatomy and connected structures.

## Methods

### Estimating neuronal connectivity

Here we describe our GLM analysis, the basis of validating connections and selecting the significance level, and the method of estimating the PSP.

#### GLMCC

To discover neuronal connections between a pair of neurons, we devise a GLM that detects short-term synaptic impacts in the CC (as schematically depicted in Fig. 1a and as real cross-correlograms of rat hippocampal data in Fig. 5b). We designed the GLMCC as

6 $$c(t)\,=\,\exp \left(a(t)\,+\,{J}_{12}f(t)\,+\,{J}_{21}f(-t)\right),$$

where $t$ is the time from the spikes of the reference neuron, and $a(t)$ represents large-scale fluctuations produced outside of the pair of neurons. ${J}_{ij}$ represents neuronal connection from the $j$th neuron to the $i$th neuron. The time profile of the synaptic interaction is modeled as $f(t)\,=\,\exp (-\frac{t\,-\,d}{\tau })$ for $t\ > \ d$ and $f(t)\,=\,0$ otherwise, where $\tau$ is the typical time scale of synaptic impact and $d$ is the transmission delay. The connection parameter ${J}_{ij}$ of our GLMCC can be derived from a model of the original interaction process between neurons (Supplementary Note 2).

Given an underlying rate $c(t)$, the probability for spikes to occur at $\{{t}_{k}\}\,=\,\{{t}_{1},{t}_{2},\cdots \ ,{t}_{N}\}$ is obtained theoretically as^62^,

7 $$p(\{{t}_{k}\}|\theta)\,=\,{\prod }_{k}c({t}_{k})\exp \left[-{\int }_{-W}^{W}c(t)\ dt\right],$$

where $\theta \,=\,\{{J}_{12},{J}_{21},a(t)\}$, representing a set of parameters that characterize $c(t)$.

To detect short-term synaptic impacts of a few ms hidden in large-scale fluctuations in the CC, we make $a(t)$ adapt to the slow part of the fluctuations. This may be done by providing a prior distribution that penalizes a large gradient of $a(t)$:

8 $$p(\theta)\propto \exp \left[-\frac{1}{\gamma}{\int}_{-W}^{W}{\left(\frac{da}{dt}\right)}^{2}\ dt\right],$$

where $\gamma$ is a hyperparameter representing the flatness of $a(t)$; $a(t)$ is nearly constant if $\gamma$ is small, or is otherwise rapidly fluctuating. We selected the hyperparameter using the ABIC (Akaike Bayesian Information Criterion)^63^ so that c(t) fits the experimental CCs, and adopted the mean value: $\gamma \,=\,5\times 1{0}^{-4}$ [ms${}^{-1}$]. For the connection parameters ${J}_{12}$ and ${J}_{21}$, we have assumed uniform priors.

The posterior distribution of a set of parameters $\theta \,=\,\{{J}_{12},{J}_{21},a(t)\}$, given the spike data $\{{t}_{k}\}$, is obtained from Bayes’ rule as

9 $$p(\theta |\{{t}_{k}\})\,=\,\frac{p(\{{t}_{k}\}|\theta )p(\theta )}{p(\{{t}_{k}\})}.$$

The parameters are determined with the maximum *a posteriori* (MAP) estimate, that is, by maximizing the posterior distribution or its logarithm:

10 $$\mathrm{log}\ {\it{p}}(\theta |\{{t}_{k}\})\,=\,{\sum }_{k}\mathrm{log}\ {\it{c}}({t}_{k})-{\int }_{-W}^{W}{\it{c}}(t)\ {\it{dt}}\\ \,-\,\frac{1}{\gamma }{\int }_{-W}^{W}{\left(\frac{\it{da}}{\it{dt}}\right)}^{2}\ {\it{dt}} + {\rm{const.}}$$

The MAP inference for $\theta \,=\,\{{J}_{12},{J}_{21},a(t)\}$ was performed efficiently using the Levenberg–Marquardt method (Supplementary Note 3).

#### Statistical test for determining connectivity

We determine the presence of a neuronal connection by disproving the null hypothesis that a connection is absent. Namely, we conclude that a connection is likely present if the estimated parameter is outside the confidence interval for the null hypothesis; otherwise, the presence of a connection is undetermined. The null hypothesis is that two neurons generate spikes at their baseline firing rates independently of each other. According to Poisson statistics, the variance of the number of spikes generated in a time interval $\Delta$ after the spike of a reference neuron is equal to its mean. The mean spike number is obtained by multiplying the intensity $c(0)$ by an interval $\Delta$,

11 $$n\,=\,c(0)\Delta .$$

Assuming that the connection $J$ is small, the average number of spikes caused by a neuronal connection during an interval $\Delta$ is approximated as

12 $$\delta n\,=\,c(0)J\tau (1\,-\,{e}^{-\Delta /\tau }).$$

The condition that the synaptic interaction produces a significant impact on the CC is $|\delta n|> {z}_{\alpha }\sqrt{n}$, where ${z}_{\alpha }$ is a threshold for the normal distribution (${z}_{\alpha }=2.58$ for $\alpha =0.01$ and ${z}_{\alpha }=3.29$ for $\alpha =0.001$). In terms of the estimated connection parameter $\hat{J}$, this condition is given as

13 $$|\hat{J}|\,> \,{z}_{\alpha }\frac{{\Delta }^{1/2}}{\tau (1-{e}^{-\Delta /\tau })}\cdot \frac{1}{{(c(0))}^{1/2}}.$$

Here, ${\Delta }^{1/2}/(\tau (1-{e}^{-\Delta /\tau }))$ on the right-hand side of this inequality is dependent on $\Delta$ but it takes the lowest value $1.57{\tau }^{-1/2}$ at $\Delta =1.26\tau$. Thus we have the following inequality:

14 $$|\hat{J}|\,> \,1.57{z}_{\alpha }{(\tau c(0))}^{-1/2}.$$

The typical duration of spike recording needed for the connectivity inference (inequality (3)) is obtained from Eq. (14) by approximating $c(0)=T{\lambda }_{{\rm{pre}}}{\lambda }_{{\rm{post}}}$, where $T$ is the total duration of recording.

Another requirement is that spike trains should contain a sufficiently large number of spikes to make a reliable inference. A typical number of spikes contained in the CC in the interaction time window is $T{\lambda }_{{\rm{pre}}}{\lambda }_{{\rm{post}}}\tau$. By requiring this to be >10, we obtain the inequality (4).

#### Selecting the significance level

Although we obtained the confidence interval of the connection parameter ${J}_{ij}$ at the given value above, the probability of assigning spurious connectivity to anatomically disconnected pairs is higher than the threshold, because spike trains are correlated. Such spurious connections or FPs may be reduced by decreasing the significance level. However, this operation may cause the vast majority of existing connections to be missed, thus producing a huge number of FNs. Thus, the significance level should be chosen so that these conflicting demands (of reducing FPs and FNs) are optimally balanced.

As we can directly count FPs and FNs in simulation data, we may select a significance level such that the performance of the inference is maximized. As a measure for assessing the performance of connectivity inference, we adopt the MCC^40^ defined as

$$\begin{array}{ccc}MCC&=&\\ &&\frac{{N}_{{\rm{T}}{\rm{P}}}{N}_{{\rm{T}}{\rm{N}}}-{N}_{{\rm{F}}{\rm{P}}}{N}_{{\rm{F}}{\rm{N}}}}{\sqrt{({N}_{{\rm{T}}{\rm{P}}}+{N}_{{\rm{F}}{\rm{P}}})({N}_{{\rm{T}}{\rm{P}}}+{N}_{{\rm{F}}{\rm{N}}})({N}_{{\rm{T}}{\rm{N}}}+{N}_{{\rm{F}}{\rm{P}}})({N}_{{\rm{T}}{\rm{N}}}+{N}_{{\rm{F}}{\rm{N}}})}},\end{array}$$

where ${N}_{{\rm{T}}{\rm{P}}}$, ${N}_{{\rm{T}}{\rm{N}}}$, ${N}_{{\rm{F}}{\rm{P}}}$, and ${N}_{{\rm{F}}{\rm{N}}}$ represent the numbers of true positive, true negative, FP, and FN connections, respectively.

Because there are excitatory and inhibitory connections, we may obtain two coefficients for individual categories. To evaluate the quality of inference in terms of a single measure, here we take the macro-average MCC that gives equal importance to these categories (Macro-average)^64^:

15 $$MCC=\frac{MC{C}_{{\rm{E}}}+MC{C}_{{\rm{I}}}}{2}.$$

In computing the coefficient for the excitatory category $MC{C}_{E}$, we classify connections as excitatory or other (disconnected and inhibitory); for the inhibitory category $MC{C}_{I}$, we classify connections as inhibitory or other (disconnected and excitatory). Here we evaluate $MC{C}_{E}$ by considering only excitatory connections of reasonable strength (EPSP $\,> \,$ 1 mV), as EPSPs distribute log-normally and there are a number of weak connections that are hard to detect in several hours.

#### Estimating PSPs from GLM connection parameters

We translate the GLM connection parameters ${J}_{ij}$ into biological PSPs ${w}_{ij}$ mV. This relation is obtained by numerically simulating a network of neurons interacting through known connections $\{{w}_{ij}\}$ and by applying the GLM to their spike trains to estimate the connection parameters $\{{J}_{ij}\}$. Regarding synaptic connections ${w}_{ij}$ for which ${J}_{ij}$ was verified in the correct signs, we assume a proportional relation as in Eq. (2):

$${J}_{ij}=a{w}_{ij}.$$

The coefficient $a$ is determined by applying regression analysis to the synthetic data. We obtained $a=0.39$ for EPSP and $1.57$ for IPSP, respectively.

When we newly estimate connection parameters ${\hat{J}}_{ij}$ from spike trains, they can be translated into PSPs using the relation:

16 $${\hat{w}}_{ij}={\hat{J}}_{ij}/a.$$

Figure 3b compares the estimated PSPs ${\hat{w}}_{ij}$ with the original PSPs values ${w}_{ij}$ of a model neural network.

In our numerical simulation, synaptic connectivity is given in terms of conductance. Thus we have to translate conductance into PSP. The translation rule is described in Supplementary Note 4 and Supplementary Fig. 8.

### Details of existing methods

Here we describe the details of the conventional CC method and the jittering method, which were compared with the present GLMCC method in estimating synaptic connectivity.

The CC method estimates the deviation in the cross-correlogram at short time-lags^16^. The synaptic connection is detected if the spike count is outside the confidence interval for a null hypothesis that two spike trains are independent stationary Poisson processes. The cross-correlogram was constructed by counting the number of spikes in an interval [−50, $+$50] ms with a bin size of $\Delta =1$ ms. The confidence interval is given by $[{\bar{n}}_{{\rm{cc}}}-{z}_{\alpha }\sqrt{{\bar{n}}_{{\rm{cc}}}},{\bar{n}}_{{\rm{cc}}}+{z}_{\alpha }\sqrt{{\bar{n}}_{{\rm{cc}}}}]$, where ${\bar{n}}_{{\rm{cc}}}={\lambda }_{{\rm{pre}}}{\lambda }_{{\rm{post}}}T\Delta$ is the expected number of spikes; ${\lambda }_{{\rm{pre}}}$ and ${\lambda }_{{\rm{post}}}$ are the firing rates of the pre- and postsynaptic neurons, respectively; and ${z}_{\alpha }$ is the threshold for the normal distribution. We have chosen the significance level $\alpha =0.01$.

The jittering method was introduced to avoid false detection caused by large fluctuations in the background cross-correlogram^20,25^. Here we adopted the parameters in the original method. Namely, we generated surrogate data sets by randomly perturbing or jittering the original data in a uniform interval of [−5,$+$5] ms to estimate a global band at an acceptance level of 99%. An excitatory or inhibitory monosynaptic connections was identified if the original cross-correlogram at a bin size of 1 ms protruded the band anywhere in the region [1, 4] ms.

### A network of HH-type neurons

We ran a numerical simulation of a network of 1000 HH-type neurons interacting through fixed synapses. Of them, 800 excitatory neurons innervate to 12.5% of other neurons with EPSPs that are log-normally distributed^34,35,37^, whereas 200 inhibitory neurons innervate randomly to 25% of other neurons with IPSPs that are normally distributed. Simulated spike trains and the connectivity matrix (EPSPs and IPSPs) are available on figshare^65^.

#### Neuron models

For excitatory pyramidal cells, we adopted HH-type models developed by Destexhe et al.^66^. The membrane potential $V$ obeys the equation:

17 $${C}_{{\rm{m}}}^{{\rm{pyr}}}\frac{dV}{dt}\,=\,-{I}_{{\rm{L}}}-{I}_{{\rm{Na}}}-{I}_{{\rm{K}}}-{I}_{{\rm{M}}}-{I}_{{\rm{tot}}},$$

where ${C}_{{\rm{m}}}^{{\rm{pyr}}}$ is the membrane capacitance, ${I}_{{\rm{L}}}={g}_{{\rm{L}}}^{{\rm{pyr}}}(V-{E}_{{\rm{L}}}^{{\rm{pyr}}})$ is the leak current, ${I}_{{\rm{Na}}}\,=\,{g}_{{\rm{Na}}}^{{\rm{pyr}}}{m}^{3}h(V\,-\,{E}_{{\rm{Na}}}^{{\rm{pyr}}})$ is the Na${}^{+}$ current, ${I}_{{\rm{K}}}\,=\,{g}_{{\rm{K}}}^{{\rm{pyr}}}{n}^{4}(V\,-\,{E}_{{\rm{K}}}^{{\rm{pyr}}})$ is the delayed-rectifier K${}^{+}$ current, ${I}_{{\rm{M}}}\,=\,{g}_{{\rm{M}}}^{{\rm{pyr}}}p(V\,-\,{E}_{{\rm{K}}}^{{\rm{pyr}}})$ is the muscarinic potassium current, and ${I}_{{\rm{tot}}}$ is the total input current from the other neurons. The gating variables $x\in \{m,h,n,p\}$ are described by the kinetic equation:

18 $$\frac{dx}{dt}\,=\,{\alpha }_{x}(V)(1\,-\,x)\,-\,{\beta }_{x}(V)x,$$

where ${\alpha }_{x}$ and ${\beta }_{x}$ are the activation and inactivation functions, respectively. The activation and inactivation functions and the parameter values are summarized in Table 2.

**Table 2:** Parameters for pyramidal neurons and interneurons

Neuron models	
${C}_{{\rm{m}}}^{{\rm{pyr}}}$, ${C}_{{\rm{m}}}^{{\rm{inh}}}$ [$\mu$F cm${}^{-2}$]	1.0, 1.0
${g}_{{\rm{L}}}^{{\rm{pyr}}}$, ${g}_{{\rm{L}}}^{{\rm{inh}}}$ [mS cm${}^{-2}$]	0.045, 0.1
${g}_{{\rm{Na}}}^{{\rm{pyr}}}$, ${g}_{{\rm{Na}}}^{{\rm{inh}}}$ [mS cm${}^{-2}$]	50.0, 112.0
${g}_{{\rm{K}}}^{{\rm{pyr}}}$, ${g}_{{\rm{M}}}^{{\rm{pyr}}}$, ${g}_{{\rm{K1}}}^{{\rm{inh}}}$, ${g}_{{\rm{K2}}}^{{\rm{inh}}}$ [${\rm{mS}}\ {{\rm{cm}}}^{-2}$]	5.0, 0.07, 0.224, 224.0
${E}_{{\rm{L}}}^{{\rm{pyr}}}$, ${E}_{{\rm{L}}}^{{\rm{inh}}}$ [mV]	−80.0, −70.0
${E}_{{\rm{Na}}}^{{\rm{pyr}}}$, ${E}_{{\rm{Na}}}^{{\rm{inh}}}$ [mV]	50.0, 55.0
${E}_{{\rm{K}}}^{{\rm{pyr}}}$, ${E}_{{\rm{K}}}^{{\rm{inh}}}$ [mV]	−90.0, −97.0
$S$ [cm${}^{2}$]	$3.5\times 1{0}^{-4}$

For inhibitory interneurons, we adopted the HH-type models developed by Erisir et al.^67^. The membrane potential $V$ obeys the equation:

19 $${C}_{{\rm{m}}}^{{\rm{inh}}}\frac{dV}{dt}=-{I}_{{\rm{L}}}-{I}_{{\rm{Na}}}-{I}_{{{\rm{K}}}_{1}}-{I}_{{{\rm{K}}}_{2}}-{I}_{{\rm{tot}}},$$

where ${C}_{{\rm{m}}}^{{\rm{inh}}}$ is the membrane capacitance, ${I}_{{\rm{L}}}\,=\,{g}_{{\rm{L}}}^{{\rm{inh}}}(V\,-\,{E}_{{\rm{L}}}^{{\rm{inh}}})$ is the leak current, ${I}_{{\rm{Na}}}\,=\,{g}_{{\rm{Na}}}^{{\rm{inh}}}{m}^{3}h(V\,-\,{E}_{{\rm{Na}}}^{{\rm{inh}}})$ is the Na${}^{+}$ current, ${I}_{{{\rm{K}}}_{1}}\,=\,{g}_{{{\rm{K}}}_{1}}^{{\rm{inh}}}{n}_{1}^{4}(V\,-\,{E}_{{\rm{K}}}^{{\rm{inh}}})$ and ${I}_{{{\rm{K}}}_{2}}={g}_{{{\rm{K}}}_{2}}^{{\rm{inh}}}{n}_{2}^{2}(V-{E}_{{\rm{K}}}^{{\rm{inh}}})$ are the delayed-rectifier K${}^{+}$ current due to Kv1.3 and Kv3.1–Kv3.2 conductance, respectively, and ${I}_{{\rm{tot}}}$ is the total input current. The gating variables $x\in \{m,h,{n}_{1},{n}_{2}\}$ follow the kinetic equation (18), with the activation and inactivation functions prescribed by the original paper^67^. The parameter values are summarized in Table 2.

#### Synaptic connections

Each neuron receives synaptic currents induced by the firing of other neurons. Excitatory synaptic currents are mediated by 2-amino-3-(5-methyl-3-oxo-1,2-oxazol-4-yl) propanoic acid (AMPA) and N-methyl-D-aspartate (NMDA) receptors, whereas inhibitory synaptic currents are mediated by $\gamma$-aminobutyric acid (GABA)-A receptors. The total input current to the $i$th neuron is given by

20 $${I}_{{\rm{tot}}}^{i} = {\sum}_{j{:} {\rm{Pyramidal}} \, {\rm{cells}}} \left({I}_{{\rm{AMPA}}}^{ij} + {I}_{{\rm{NMDA}}}^{ij} \right) + {\sum }_{j{:}{\rm{Interneurons}}} {I}_{{\rm{GABA}}}^{ij}+{I}_{\rm{bg}}.$$

where ${I}_{{\rm{AMPA}}}^{ij}$, ${I}_{{\rm{NMDA}}}^{ij}$, and ${I}_{{\rm{GABA}}}^{ij}$, respectively represent the synaptic currents given by the AMPA, NMDA, and GABA receptors, and ${I}_{{\rm{bg}}}$ represents the background current.

For AMPA-mediated current, we adopted the depressing synapse model proposed by Tsodyks et al.^44^

21 $${I}_{{\rm{AMPA}}}^{ij}\,=\,{g}_{{\rm{AMPA}}}^{ij}{w}_{j}(t)({V}_{i}\,-\,{E}_{{\rm{AMPA}}}),$$

22 $$\begin{array}{ccc}{\tau }_{{\rm{ina}}}^{{\rm{AMPA}}}\frac{d{w}_{j}(t)}{dt}&=&-{w}_{j}(t)\\ &&+\,{U}_{{\rm{AMPA}}}{r}_{j}(t){\sum }_{k}\delta (t-{t}_{k}^{j}-{d}_{{\rm{AMPA}}}),\end{array}$$

23 $${\tau }_{{\rm{rec}}}^{{\rm{AMPA}}}\frac{d{r}_{j}(t)}{dt}\,=\,-{r}_{j}(t)\,+\,1\,-\,{w}_{j}(t),$$

where ${g}_{{\rm{AMPA}}}^{ij}$ is the maximal AMPA conductance, ${V}_{i}$ is the membrane potential of the postsynaptic neuron, ${t}_{k}^{j}$ is the $k$th spike time of the presynaptic neuron, and ${d}_{{\rm{AMPA}}}$ is the synaptic conduction delay. For each connection, the conduction delay is drawn from a uniform distribution between 0 and 2 ms. ${w}_{j}$ and ${r}_{j}$ represent the fraction of synaptic resources in the effective and recovered states, respectively. The AMPA parameter values are summarized in Table 3.

**Table 3:** Parameters for synaptic currents and background inputs

Synaptic current	
${E}_{{\rm{AMPA}}}$, ${E}_{{\rm{NMDA}}}$, ${E}_{{\rm{GABA}}}$ [mV]	0.0, 0.0, −75.0
${\tau }_{{\rm{ina}}}^{{\rm{AMPA}}}$, ${\tau }_{{\rm{ina}}}^{{\rm{GABA}}}$ [ms]	2.7, 10.0
${\tau }_{{\rm{rec}}}^{{\rm{AMPA}}}$, ${\tau }_{{\rm{rec}}}^{{\rm{GABA}}}$ [ms]	500, 500
${U}_{{\rm{AMPA}}}$, ${U}_{{\rm{GABA}}}$	0.25, 0.25
${\alpha }_{{\rm{NMDA}}}$, ${\beta }_{{\rm{NMDA}}}$ [ms${}^{-1}$]	0.5, 0.007

Background input current	
${g}_{{\rm{e,0}}}$, ${g}_{{\rm{i,0}}}$ [nS]	10.8, 51.3
${\sigma }_{{\rm{e}}}$, ${\sigma }_{{\rm{i}}}$ [nS]	2.85, 6.26
${\tau }_{{\rm{e}}}$, ${\tau }_{{\rm{i}}}$ [ms]	2.7, 10.5

For NMDA-mediated current, we adopted the first-order kinetic equation proposed by Destexhe et al.^68^

24 $${I}_{{\rm{NMDA}}}^{ij}={g}_{{\rm{NMDA}}}^{ij}{r}_{j}(t)f({V}_{i})({V}_{i}-{E}_{{\rm{NMDA}}}),$$

25 $$\begin{array}{ccc}\frac{d{r}_{j}(t)}{dt}&=&{\alpha }_{{\rm{NMDA}}}T(t-{t}_{{\rm{pre}}}-{d}_{{\rm{NMDA}}})(1-{r}_{j}(t))\\ &&-{\beta }_{{\rm{NMDA}}}{r}_{j}(t),\end{array}$$

26 $$f({V}_{i})={\left(1.0+0.28[{{\rm{Mg}}}^{2+}]{e}^{-0.062{V}_{i}}\right)}^{-1},$$

where [Mg${}^{2+}$] = 1.0 mM is the extracellular magnesium concentration, ${t}_{{\rm{pre}}}$ is the last spike time of the presynaptic neuron, ${d}_{{\rm{NMDA}}}$ is the conduction delay drawn from a uniform distribution between 0 and 2 ms, and $T(t)$ represents the transmitter concentration in the cleft. When a spike occurs in a presynaptic neuron, a transmitter pulse is induced such that $T(t)\,=\,1$ mM for a short period (1 ms) and the concentration returns to $T(t)\,=\,0$. The NMDA parameter values are summarized in Table 3.

For GABA-A-mediated current, we adopted the depressing synapse model proposed by Tsodyks et al.^44^

27 $${I}_{{\rm{GABA}}}^{ij}={g}_{{\rm{GABA}}}^{ij}{w}_{j}(t)({V}_{i}-{E}_{{\rm{GABA}}}),$$

28 $$\begin{array}{ccc}&&{\tau }_{{\rm{ina}}}^{{\rm{GABA}}}\frac{d{w}_{j}(t)}{dt}=-{w}_{j}+\\ &&\,\,\,\,\,\,{U}_{{\rm{GABA}}}{r}_{j}(t){\sum }_{k}\delta (t-{t}_{k}^{j}-{d}_{{\rm{GABA}}}),\end{array}$$

29 $${\tau }_{{\rm{rec}}}\frac{d{r}_{j}(t)}{dt}=-{r}_{j}(t)+1-{w}_{j}(t).$$

where ${d}_{{\rm{GABA}}}$ is the conduction delay drawn from a uniform distribution between 1 and 3 ms. The GABA parameter values are summarized in Table 3.

We ran a simulation of a network consisting of 800 pyramidal neurons and 200 interneurons interconnected with a fixed strength. Each neuron receives 100 excitatory inputs randomly selected from 800 pyramidal neurons and 50 inhibitory inputs selected from 200 interneurons.

The AMPA conductance (${g}_{{\rm{AMPA}}}^{ij}$) is drawn independently from a log-normal distribution^34,35^

30 $$P(x)\,=\,\frac{1}{\sqrt{2\pi }\sigma x}\exp \left(-\frac{{(\mathrm{log}\,{\it{x}}\,-\,\mu )}^{2}}{2{\sigma }^{2}}\right),$$

where $\mu \,=\,-3.37$ and $\sigma \,=\,1.3$ are the mean and SD of the natural logarithm of the AMPA conductance. The NMDA and GABA conductances (${g}_{{\rm{NMDA}}}^{ij}$ and ${g}_{{\rm{GABA}}}^{ij}$) are sampled from the normal distribution

31 $$P(x)=\frac{1}{\sqrt{2\pi }\sigma }\exp \left(-\frac{{(x-\mu )}^{2}}{2{\sigma }^{2}}\right),$$

where $\mu$ and $\sigma$ are the mean and SD of the conductances. Parameters are ${\mu }_{{\rm{NMDA}}}\,=\,8.5\times 1{0}^{-4}\ {\rm{mS}}\ {{\rm{cm}}}^{-2}$, ${\sigma }_{{\rm{NMDA}}}\,=\,8.5\times 1{0}^{-5}\ {\rm{mS}}\ {{\rm{cm}}}^{-2}$ and ${\mu }_{{\rm{GABA}}}\,=\,0.34\ {\rm{mS}}\ {{\rm{cm}}}^{-2}$, ${\sigma }_{{\rm{GABA}}}\,=\,0.27\ {\rm{mS}}\ {{\rm{cm}}}^{-2}$ for the NMDA and GABA conductance, respectively. If the sampled value is less than zero, the conductance is resampled from the same distribution.

Because our model network is smaller than real cortical networks, where each neuron receives inputs from the order of 1000 neurons, we added a background current to represent inputs from many neurons, as previously done by Destexhe et al.^69^. The background current is given as the sum of excitatory and inhibitory inputs:

32 $${I}_{{\rm{bg}}}\,=\,{g}_{{\rm{e}}}(t)(V\,-\,{E}_{{\rm{AMPA}}})\,+\,{g}_{{\rm{i}}}(t)(V\,-\,{E}_{{\rm{GABA}}}),$$

where the total excitatory and inhibitory conductance ${g}_{{\rm{e,i}}}(t)$ obey the Ornstein–Uhlenbeck process^70^, representing random bombardments from a number of neurons.

33 $$\frac{d{g}_{x}}{dt}\,=\,-\frac{{g}_{x}(t)\,-\,{g}_{x,0}}{{\tau }_{x}}\,+\,\sqrt{\frac{2{\sigma }_{x}^{2}}{{\tau }_{x}}}\xi (t),$$

where $x$ represents excitatory (e) or inhibitory (i), ${g}_{x,0}$ and ${\sigma }_{x}$ are the asymptotic mean and SD of the conductance, ${\tau }_{x}$ is the synaptic time constant, and $\xi (t)$ is the Gaussian white noise with zero mean and unit variance. Parameters for the background inputs are summarized in Table 3.

Simulation codes were written in C++ and parallelized with OpenMP framework. Simulations were conducted on a computer with Intel Xeon Processors E5-2650v2. The time step was 0.01 ms for excitatory (pyramidal) neurons and 0.001 ms for inhibitory (inter) neurons. The neural activity was simulated up to 10,000 s.

### Experimental data

Spike trains were recorded from the hippocampal area of a rat, while it was exploring an open square field. Experimental procedures, data collection, and spike sorting are as described in detail in Mizuseki et al.^51^. All protocols were approved by the Institutional Animal Care and Use Committees of Rutgers University and New York University. Hippocampal principal cells and interneurons were separated on the basis of their waveforms, autocorrelograms, and mean firing rates^49–51^.

### Reporting summary

Further information on research design is available in the Nature Research Reporting Summary linked to this article.

## Supplementary information

Supplementary Information

Reporting Summary

Source Data

Source Data

## Data Availability

The source data underlying Figs. 2–7 are provided as a Source Data file. Simulated data generated by a network of 1,000 Hodgkin–Huxley neurons has been deposited in figshare^65^ (10.6084/m9.figshare.9637904). All experimental data used in this paper can be found in hc-3 data sets at CRCNS^48^ (CRCNS.org. 10.6080/K09G5JRZ).

## Code availability

A ready-to-use version of the web application, the source code, and example data sets are available at our website, http://www.ton.scphys.kyoto-u.ac.jp/%7Eshino/GLMCC and are also hosted publicly on github, accessible via https://github.com/NII-Kobayashi. Simulation codes of a large network of LIF neurons are available on ModelDB (https://senselab.med.yale.edu/modeldb/ShowModel.cshtml?model=258807). Simulation codes of a network of HH neurons are available upon request from the corresponding author.
