# Supplementary material for: Reconstructing neuronal circuitry from parallel spike trains
Source: Nat Commun. 2019 Oct 2;10:4468. doi: 10.1038/s41467-019-12225-2 (PMC6775109; doi:10.1038/s41467-019-12225-2)
Supplement: Supplementary file 1 — Supplementary Information [file 41467_2019_12225_MOESM1_ESM.pdf]

Supplementary Information

Ryota Kobayashi, Shuhei Kurita, Anno Kurth, Katsunori Kitano, Kenji Mizuseki, Markus Diesmann, Barry J. Richmond, and Shigeru Shinomoto[a)]

(Dated: 20 August 2019)

[a)]Electronic mail: shinomoto.shigeru.6e@kyoto-u.ac.jp

Supplementary Figures

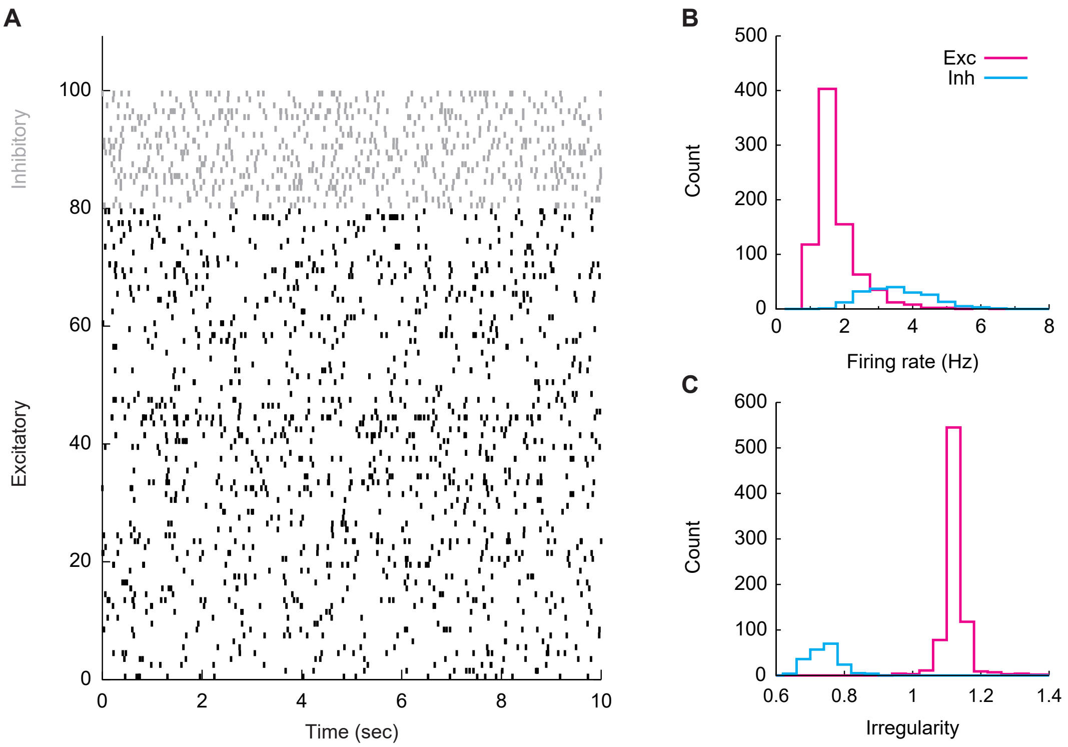

Supplementary Figure 1. **Firing characteristics of simulation model.** The network of Hodgkin-Huxley type neurons exhibit the irregular firing and the skewed distribution of firing rates, which are consistent with the chaotic dynamics of the balanced network models[1,2]. (A) Raster plot of the firing activity of 100 neurons (sampled from 1,000 neurons of Hodgkin-Huxley type neurons) for 10 seconds of biological time (black: Excitatory neurons; gray: Inhibitory neurons). (B) Histograms of the firing rates of excitatory and inhibitory neurons. (C) Firing irregularity measured in terms of the local variation of the interspike intervals Lv[3].

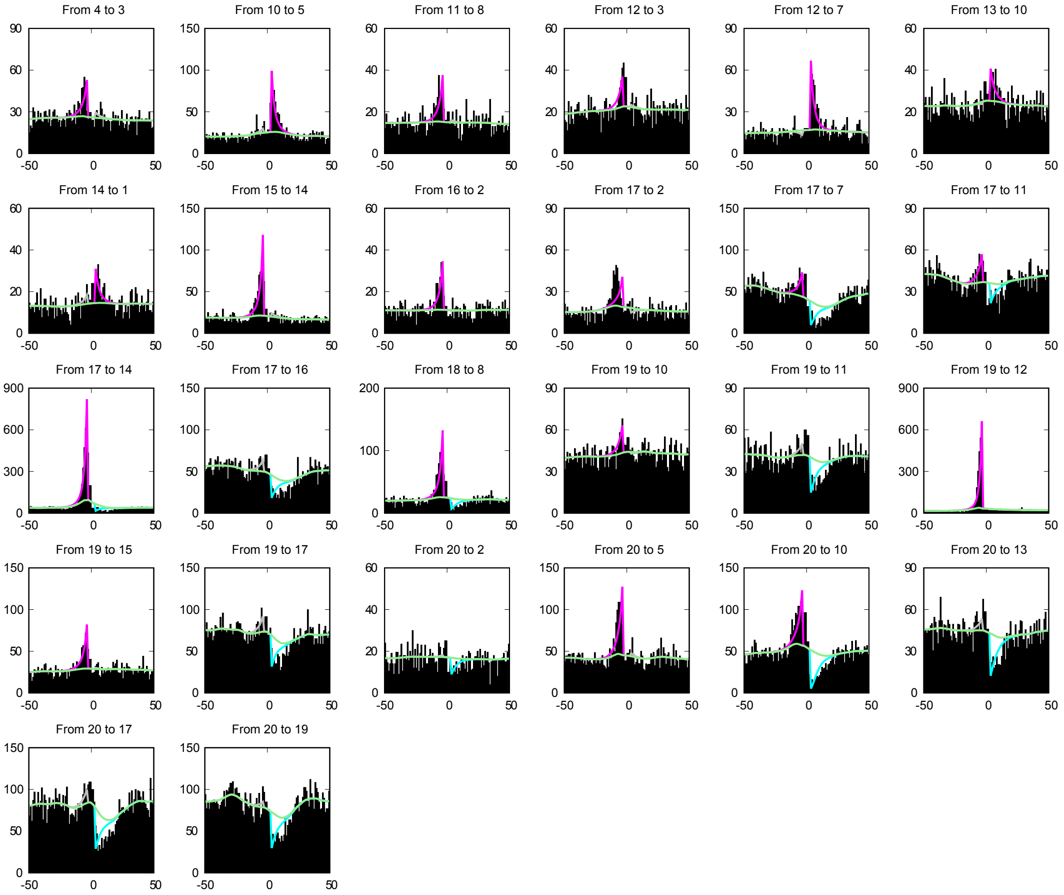

Supplementary Figure 2. GLMCC fitting to cross-correlograms for detected connections in synthetic data of HH neurons (90 min in Fig. 3A). The slow part of the GLM adapted to the data is depicted as a light green line. The coupling filter is separately depicted in magenta, cyan, or gray, for the excitatory, inhibitory, or undetermined connection, respectively.

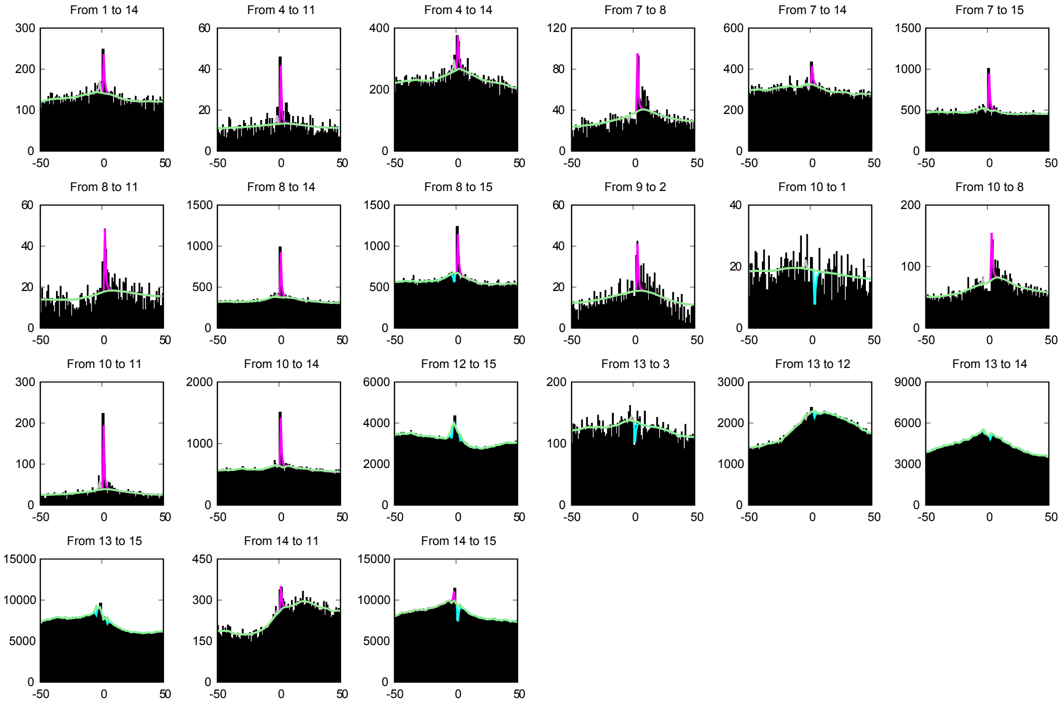

Supplementary Figure 3. **GLMCC fitting to cross-correlograms for detected connections in CA1 data (90 min in Fig. 5A).** The slow part of the GLM adapted to the data is depicted as a light green line. The coupling filter is separately depicted in magenta, cyan, or gray, for the excitatory, inhibitory, or undetermined connection, respectively.

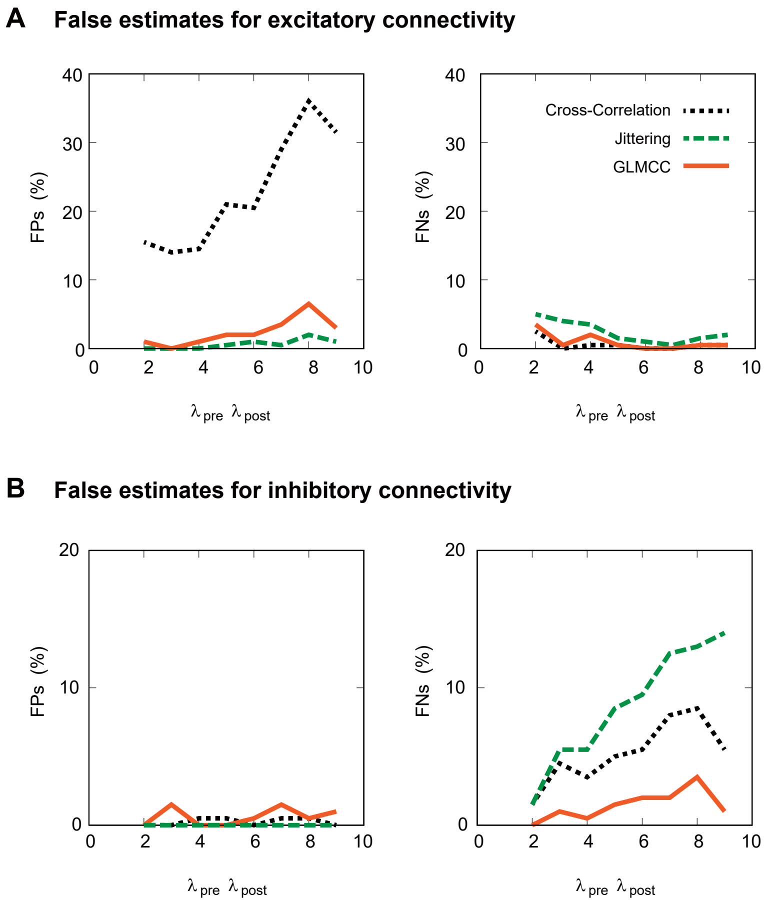

Supplementary Figure 4. Dependence of the number of estimation errors on the firing rate. The number of false positives (FPs) and false negatives (FNs) is counted for pairs of neurons whose product of firing rates $\lambda_{\text{pre}}\lambda_{\text{post}}$ is close to a given value (deviation less than 10%). The jittering method rarely produces FPs but instead it produces a large number of FNs. Our GLMCC consistently produces a smaller number of errors in total than the two other estimation methods. (A) Excitatory connectivity: FPs represent directed links that were mistakenly assigned as excitatory, whereas FNs represent excitatory connections that were assigned as disconnected or inhibitory. (B) Inhibitory connectivity: FPs represent directed links that were mistakenly assigned as inhibitory, whereas FNs represent inhibitory connections that were assigned as disconnected or excitatory.

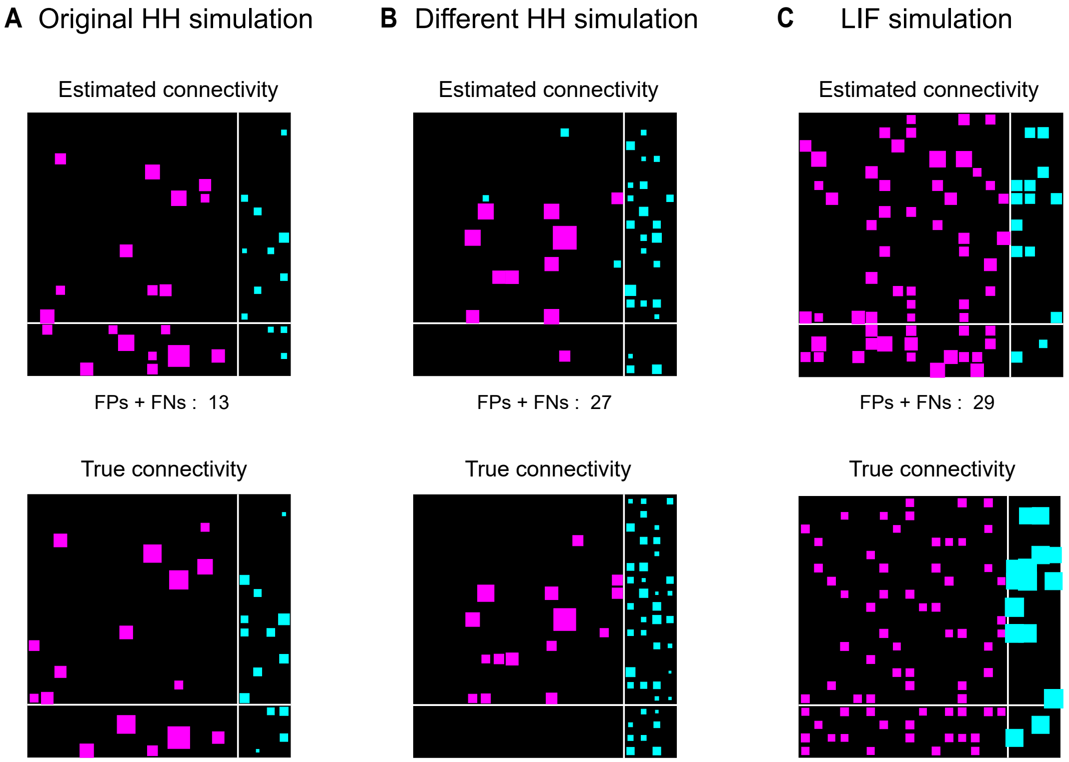

Supplementary Figure 5. **Estimating connectivity for several different data sets.** (A) The original data (90 min in Fig. 3A). (B) Data obtained with a different inhibition level. (C) Data obtained from Leaky Integrate-and-Fire neurons[4,5]. We confirmed that the method estimates the connectivity accurately for these data as well.

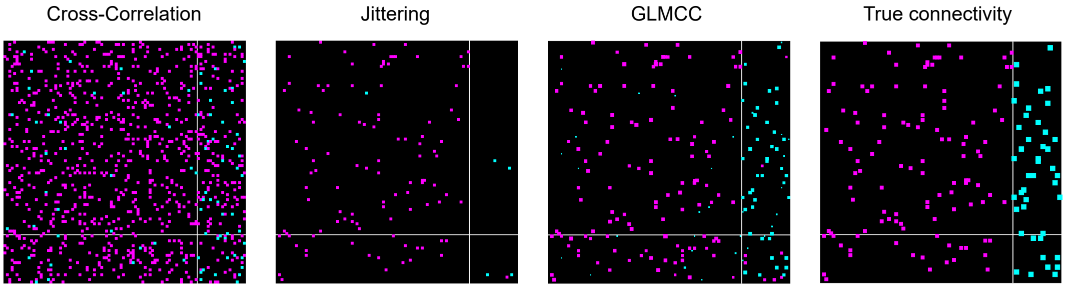

Supplementary Figure 6. **Connections estimated from a network of 10,000 LIF neurons.** Connection matrices estimated with the conventional cross-correlation method, the jittering method, and our GLMCC method are displayed in reference to the true connectivity of the simulation data. In this simulation, connections of equal strength are wired randomly among neurons (See Supplementary Note 1 for details). 100 neurons (80 excitatory and 20 inhibitory neurons) are randomly sampled from 10,000 LIF neurons. Average number of excitatory inputs to each neuron: 100. Observation time windows: 5,400 s (90 min).

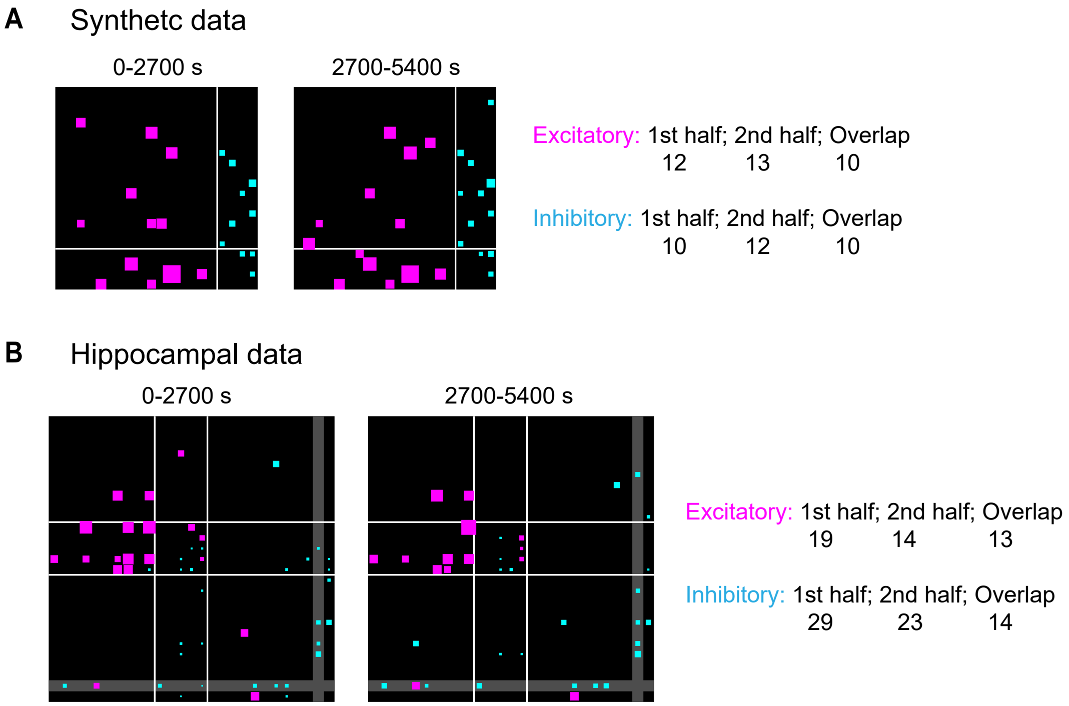

Supplementary Figure 7. Neuronal connections estimated for the first and second halves of the recording interval. There was significant overlap between the first and second halves, not only for the simulation data, but also for the experimental data. (A) Connection matrices estimated from synthetic data. (B) Connection matrices estimated from the rat hippocampal data.

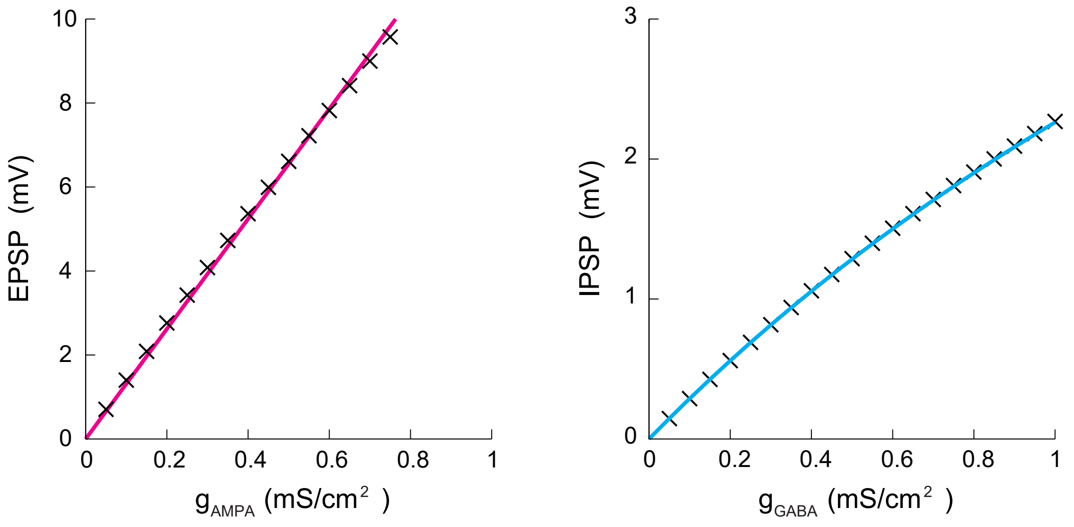

Supplementary Figure 8. Relationship between PSP and synaptic conductance. Magenta and cyan lines represent the fittings given in equations (S9) in Supplementary note 4 for EPSP and IPSP, respectively.

Supplementary Note 1: Large-scale simulation using the NEST simulator

We conducted a large-scale simulation of a cortical network consisting of 10,000 neurons (See Supplementary Tables 1, 2, and 3 for details). The network is defined by two neural populations representing excitatory (8,000 neurons) and inhibitory (2,000 neurons) cells. Each population consists of leaky integrate-and-fire model neurons with exponential synaptic currents, which are randomly connected with a fixed number of outgoing connections termed outdegree. The outdegree of an inhibitory neuron is twice as large as of an excitatory one. Each neuron receives Poissonian background spike trains[2,4,6] as well as an injected external current to maintain network activity in the asynchronous irregular regime. Parameters of the single neuron model are adopted from Zaytsev et al.,[4] (See Supplementary Table 2).

All simulations are carried out with the NEST simulation tool[7] using a grid constrained solver and a computation step size: 0.1 ms on a compute cluster with 32 nodes each equipped with 2 Intel Xeon E5-2680v3 processors (hyperthreading enabled) and interconnected by a 36-port QSFP QDR InfiniBand switch. An open source implementation of the network simulation will be available at ModelDB (https://senselab.med.yale.edu/modeldb/).

Supplementary Note 2: Derivation of the GLMCC from a two-body GLM

The GLMCC (equation 6 in the main text) can be derived from a two-body GLM describing individual neurons interacting with each other. This is given by the firing rates of two neurons, $\lambda_1(t)$ and $\lambda_2(t)$:

$$\lambda_1(t) = \exp(u_1(t) + J_{12}s_2(t)), \tag{S1}$$

$$\lambda_2(t) = \exp(u_2(t) + J_{21}s_1(t)), \tag{S2}$$

where $u_i(t)$ represents extrinsic fluctuations for each neuron mediated through many marginal neurons. J_{ij} is the neuronal connection from the jth neuron to the ith neuron. $s_j(t)$ represents a temporal profile of monosynaptic impact:

$$s_j(t) = \sum_k f(t - t_k^j), \tag{S3}$$

where t_k^j is the time of the kth spike of the jth neuron. The monosynaptic interaction of the timescale τ is modelled here with a fixed time profile: $f(t) = \exp(-\frac{t-d}{\tau})$ for $t > d$ and $f(t) = 0$ otherwise, where d is the synaptic transmission delay.

If the precise time profiles of extrinsic fluctuations $u_1(t)$ and $u_2(t)$ are known, the mutual interactions J_{12} and J_{21} may be estimated by fitting equations (S1) and (S2) to a pair of spike trains. Detailed information for $u_1(t)$ and $u_2(t)$ may be available in some initial sensory systems. In an analysis of retinal neural networks, information on optical stimuli was efficiently utilized to estimate neuronal connectivity[8]. However, in cortical networks with denser connectivity, the temporal fluctuations may be induced not only extrinsically but also intrinsically and spontaneously because of mutual excitation between neurons[9,10]. Thus, fluctuating inputs to individual neurons $u_1(t)$ and $u_2(t)$ are generally unavailable. Though it is in principle possible to estimate external stimuli from the spike trains themselves, the temporal resolution cannot be finer than the typical inter-spike interval. This means that the influence of brain waves in subsecond time period cannot be estimated from spike trains of a few Hz.

Nevertheless, systematic fluctuations in subsecond periods are actually observed in CC, as demonstrated in Fig. 5B. This occurs because the CC is made by piling up spikes of one neuron that occurred in the neighbourhood of spikes of another neuron; accordingly, the firing rate of one neuron is in practice multiplied by the total number of spikes of another

neuron. To make the most of this information, we derive another GLM that may adapt to the CC data.

A CC of spike trains generated from the underlying rates (S1) and (S2) is given as

$$c(t) = \overline{\lambda_i(t+s)\lambda_j(s)}, \tag{S4}$$

where the over-line $\overline{\cdots}$ represents the time-average over s. By separating the time dependent fluctuations as $u_i(t) = u_i^0 + \delta u_i(t)$ and ignoring the higher order terms in the cumulant of CC, we obtain the GLMCC (equation(S5)):

$$c(t) = \exp\left(a(t) + J_{ij} f(t) + J_{ji} f(-t)\right), \tag{S5}$$

where $a(t)$ represents the fluctuation induced by the external stimuli:

$$a(t) = u_i^0 + u_j^0 + \overline{\delta u_i(t+s)\delta u_j(s)}. \tag{S6}$$

Thus $a(t)$ represents a large-scale modulation of the CC caused extrinsically and carried out by marginal neurons. The monosynaptic impacts J_{ij} and J_{ji} originally defined in the two-body GLM (equations (S1) and (S2)) appear in GLMCC (equation (S5)).

Supplementary Note 3: The Levenberg-Marquardt method

The Levenberg-Marquardt (LM) method, which interpolates between the Newton method and the gradient descent method works efficiently to maximize a function. Here, we apply this method to maximizing the log posterior distribution function $\log p(\theta) \equiv \log p(\theta|\{t_k\})$ defined by the equation (10). The LM algorithm is given as

$$\theta_{k+1} = \theta_k - \left[H(\log p(\theta_k)) + c_{\mathrm{LM}}\mathrm{diag}\left(H(\log p(\theta_k))\right)\right]^{-1} \nabla \log p(\theta), \tag{S7}$$

where θ_k is the kth iteration parameters, $\nabla \log p$ and $H(\log p)$ are the gradient and Hessian, respectively, and $\mathrm{diag}(H)$ is a matrix consisting of the diagonal element of the Hessian matrix. If the posterior distribution $\log p(\theta_{k+1})$ increases with the iteration rule (S7), θ is updated and the parameter c_{LM} is multiplied by η; otherwise, θ is not updated but c_{LM} is multiplied by η^{-1}. We set the initial value $c_{\mathrm{LM}} = 10^{-4}$ and $\eta = 0.1$ and repeated the iteration until $\log p(\theta_{k+1}) - \log p(\theta_k) < 10^{-4}$. Because the gradient and Hessian are obtained analytically[4], one iteration (S7) requires only the computation of the order of $O(N)$, where N is the number of spikes contained in the CC.

A set of parameters of our GLMCC, θ, consists of J_{12}, J_{21}, and $\{a(t)\}$. The function $\{a(t)\}$ is treated as a set of values $\{a(t_1), a(t_2), \cdots, a(t_M)\}$ discretized at 1 [ms] in an interval of $[-W, W] = [-50, 50]$ [ms], where $M = 100$ is the number of intervals.

Supplementary Note 4: Translating synaptic conductance into postsynaptic potential (PSP)

Our numerical simulation was performed on a network of Hodgkin−Huxley type neurons whose synaptic connections are given in terms of conductance. Even with a fixed conductance, PSP may vary depending on the voltage of the postsynaptic neuron as well as the firing activity of the presynaptic neuron. To represent conductance in terms of PSP, we must estimate the typical level of PSP induced by the given conductance level. This was done by setting the membrane voltage of the postsynaptic neuron at the resting level and the firing rate of the presynaptic neuron at 3 Hz.

The typical excitatory PSP (EPSP) and inhibitory PSP (IPSP) were calculated using a neuron model:

$$\frac{dV}{dt} = -g_{\text{eff}}(V - E_{\text{eff}}) + I_{\text{AMPA/GABA}}(t), \tag{S8}$$

where $g_{\text{eff}} = 0.27$ mS/cm^2 and $E_{\text{eff}} = -67$ mV are the effective conductance and resting voltage, respectively, and $I_{\text{AMPA/GABA}}(t)$ is a synaptic current mediated by either the AMPA or GABA receptor, respectively representing excitatory or inhibitory synapses. This model is known to reproduce the sub-threshold voltage trace of HH-type neurons and cortical neurons recorded *in vitro*[11,12]. Typical EPSP (mV) and IPSP (mV) values were well-fitted by the following equations (Supplementary Fig. 8):

$$\text{EPSP} = 13.1\, g_{\text{AMPA}}, \ \text{IPSP} = \frac{9.5\, g_{\text{GABA}}}{3.2 + g_{\text{GABA}}}, \tag{S9}$$

On the basis of these empirical relations, the model conductances g_{AMPA} and g_{GABA} are translated into EPSP and IPSP, respectively.

Supplementary Table 1. Model description after Nordlie et al.,[13]: Large-scale cortical network

**Model Summary**	
Populations	2 populations, one of which consisting of excitatory, the other of inhibitory neurons
Connectivity	random connectivity with fixed outdegree for each neuron
Neuron model	leaky integrate-and-fire (LIF), fixed absolute refractory period
Synapse model	exponential post-synaptic current
Input	independent homogenous Poisson spike trains, constant injected current
Measurement	spiking activity
**Populations**	
Population size	$8,000$ excitatory, $2,000$ inhibitory neurons
**Neuron and synapse model**	
Subthreshold dynamics	$\frac{dV}{dt} = -\frac{V}{\tau_m} + \frac{I(t)}{C_m}, \quad I_{\text{syn}} = we^{-(t-t^*-d)/\tau_{\text{syn}}}\theta(t-t^*-d).$
Spiking	If $V(t-) < V_{\text{th}}$ and $V(t+) \geq V_{\text{th}}$,
	1. Set $t^* = t$ and $V(t) = V_{\text{res}}$, and 2. Emit spike with time stamp t^*.
**Connectivity**	
Type	fixed outdegree,
	i.e. for each neuron number of outdegree target-neurons drawn with replacement
Weights	fixed excitatory and inhibitory weights
Delays d	fixed delay

Supplementary Table 2. Parameters: Large-scale cortical network

**Neuron parameters**		
τ_m	20 ms	membrane time constant
τ_r	2 ms	absolute refractory period
τ_s	0.5 ms	postsynaptic current time constant
C_m	0.45 pF	membrane capacity
V_{res}	0 mV	reset potential
V_{th}	20 mV	firing threshold
**Synapse parameters**		
w_{ex}	2 mV	synaptic efficacy in maximal EPSP value
g	7.5	relative inhibitory synpatic efficacy
d	2 ms	synaptic delay

Supplementary Table 3. Variants of outdegree with corresponding inputs; average incoming excitatory conenctions to a neuron are $10, 20, 50, 100$, and 200

Outdegree of excitatory neurons	12	25	62	125	250
Outdegree of inhibitory neurons	24	50	124	250	500
Poisson input to excitatory neurons in Hz	550	550	550	550	550
Poisson input to inhibitory neurons in Hz	600	600	600	600	650
Injected current to excitatory neurons in pA	-0.32	-0.28	-0.19	-0.06	0.31
Injected current to inhibitory neurons in pA	-0.32	-0.28	-0.19	-0.06	0.3

REFERENCES

[1]C. Van Vreeswijk and H. Sompolinsky, "Chaos in neuronal networks with balanced excitatory and inhibitory activity," Science **274**, 1724–1726 (1996).

[2]T. C. Potjans and M. Diesmann, "The cell-type specific cortical microcircuit: relating structure and activity in a full-scale spiking network model," Cerebral cortex **24**, 785–806 (2014).

[3]S. Shinomoto, K. Shima, and J. Tanji, "Differences in spiking patterns among cortical neurons," Neural Comput. **15**, 2823–2842 (2003).

[4]Y. V. Zaytsev, A. Morrison, and M. Deger, "Reconstruction of recurrent synaptic connectivity of thousands of neurons from simulated spiking activity," J. Comput. Neurosci. **39**, 77–103 (2015).

[5]Y. V. Zaytsev, A. Morrison, and M. Deger, "mletools: simulated spiking activity [data set]," Zenodo (2015), `http://doi.org/10.5281/zenodo.17662`.

[6]N. Brunel, "Dynamics of sparsely connected networks of excitatory and inhibitory spiking neurons," Journal of computational neuroscience **8**, 183–208 (2000).

[7]M.-O. Gewaltig and M. Diesmann, "Nest (neural simulation tool)," Scholarpedia **2**, 1430 (2007).

[8]J. W. Pillow, J. Shlens, L. Paninski, A. Sher, A. M. Litke, E. Chichilnisky, and E. P. Simoncelli, "Spatio-temporal correlations and visual signalling in a complete neuronal population," Nature **454**, 995 (2008).

[9]S. Ostojic, "Two types of asynchronous activity in networks of excitatory and inhibitory spiking neurons," Nat. Neurosci. **17**, 594 (2014).

[10]T. Onaga and S. Shinomoto, "Emergence of event cascades in inhomogeneous networks," Sci. Rep. **6**, 33321 (2016).

[11]R. Jolivet, R. Kobayashi, A. Rauch, R. Naud, S. Shinomoto, and W. Gerstner, "A benchmark test for a quantitative assessment of simple neuron models," J. Neurosci. Methods **169**, 417–424 (2008).

[12]R. Kobayashi and K. Kitano, "Impact of slow k^+ currents on spike generation can be described by an adaptive threshold model," J. Comput. Neurosci. **40**, 347–362 (2016).

[13]E. Nordlie, M.-O. Gewaltig, and H. E. Plesser, "Towards reproducible descriptions of neuronal network models," PLoS Comput. Biol. **5**, e1000456 (2009).
