# Supplementary material for: Reconstructing neuronal circuitry from parallel spike trains
Source: Nat Commun. 2019 Oct 2;10:4468. doi: 10.1038/s41467-019-12225-2 (PMC6775109; doi:10.1038/s41467-019-12225-2)
Supplement: Supplementary file 2 — Reporting Summary [file 41467_2019_12225_MOESM2_ESM.pdf]

natureresearch

Corresponding author(s): Shigeru Shinomoto

Last updated by author(s): Jul 2, 2019

Reporting Summary

Nature Research wishes to improve the reproducibility of the work that we publish. This form provides structure for consistency and transparency in reporting. For further information on Nature Research policies, see Authors & Referees and the Editorial Policy Checklist .

Statistics

For all statistical analyses, confirm that the following items are present in the figure legend, table legend, main text, or Methods section.

n/a	Confirmed	
☐	☒	The exact sample size (*n*) for each experimental group/condition, given as a discrete number and unit of measurement
☐	☒	A statement on whether measurements were taken from distinct samples or whether the same sample was measured repeatedly
☐	☒	The statistical test(s) used AND whether they are one- or two-sided<br>*Only common tests should be described solely by name; describe more complex techniques in the Methods section.*
☒	☐	A description of all covariates tested
☐	☒	A description of any assumptions or corrections, such as tests of normality and adjustment for multiple comparisons
☐	☒	A full description of the statistical parameters including central tendency (e.g. means) or other basic estimates (e.g. regression coefficient) AND variation (e.g. standard deviation) or associated estimates of uncertainty (e.g. confidence intervals)
☐	☒	For null hypothesis testing, the test statistic (e.g. *F*, *t*, *r*) with confidence intervals, effect sizes, degrees of freedom and *P* value noted<br>*Give P values as exact values whenever suitable.*
☐	☒	For Bayesian analysis, information on the choice of priors and Markov chain Monte Carlo settings
☒	☐	For hierarchical and complex designs, identification of the appropriate level for tests and full reporting of outcomes
☒	☐	Estimates of effect sizes (e.g. Cohen's *d*, Pearson's *r*), indicating how they were calculated

Our web collection on statistics for biologists contains articles on many of the points above.

Software and code

Policy information about availability of computer code

Data collection	The simulation data of the cortical network was generated by using C++ codes for Hodgkin-Huxley network and NEST simulator (Gewaltig and Diesmann, 2007) for Large scale LIF network. For hippocampal data, neurophysiological signals were amplified, bandpass-filtered, and acquired continuously on a 128-channel DataMax system (16-bit resolution; RC Electronics).
Data analysis	We reconstructed synaptic connectivity from spike trains of cortical neurons by using C codes with CLAPACK package for numerical linear algebra and GSL package for special function. For hippocampal data, spike sorting was performed automatically, using KlustaKwik (http://klustawik.sourceforge.net, Harris et al., 2000), followed by manual adjustment of the clusters (using “Klusters” software package; http://klusters.sourceforge.net, Hazan et al., 2006).

For manuscripts utilizing custom algorithms or software that are central to the research but not yet described in published literature, software must be made available to editors/reviewers. We strongly encourage code deposition in a community repository (e.g. GitHub). See the Nature Research guidelines for submitting code & software for further information.

Data

Policy information about availability of data

All manuscripts must include a data availability statement. This statement should provide the following information, where applicable:

- Accession codes, unique identifiers, or web links for publicly available datasets
- A list of figures that have associated raw data
- A description of any restrictions on data availability

Hippocampal data used in our study is available from CRCNS (hc3 in Mizuseki et al., 2013). Synthetic and Hippocampal data for generating Figure 5 and 6 will be available from Source Data in the paper.

Field-specific reporting

Please select the one below that is the best fit for your research. If you are not sure, read the appropriate sections before making your selection.

☒ Life sciences ☐ Behavioural & social sciences ☐ Ecological, evolutionary & environmental sciences

For a reference copy of the document with all sections, see nature.com/documents/nr-reporting-summary-flat.pdf

Life sciences study design

All studies must disclose on these points even when the disclosure is negative.

Sample size	The sample size was sufficiently large, i.e., 1,000 neurons of Hodgkin-Huxley network, 100 neurons recorded from a Large scale LIF network, and 80 neurons from rat hippocampus (Mizuseki et al., 2009).
Data exclusions	We analyzed all the simulated data sets (Hodgkin-Huxley network and Large scale LIF network). For hippocampal data, we analyze the firing activity of the neurons whose firing rate is enough large (>0.5 Hz) for reconstructing synaptic connectivity.
Replication	Superiority of our proposed method to other existing methods was confirmed by analyzing three simulated data sets, that is, Hodgkin-Huxley network (see Method), LIF network (Zaytsev et al., 2015), and Large scale LIF network (see Supplementary Note 5).
Randomization	This is not relevant to our study because the experiment focused on the computational role of entorhinal-hippocampal system.
Blinding	This is not relevant to our study (See above).

Reporting for specific materials, systems and methods

We require information from authors about some types of materials, experimental systems and methods used in many studies. Here, indicate whether each material, system or method listed is relevant to your study. If you are not sure if a list item applies to your research, read the appropriate section before selecting a response.

Materials & experimental systems

n/a	Involved in the study	
☒	☐	Antibodies
☒	☐	Eukaryotic cell lines
☒	☐	Palaeontology
☐	☒	Animals and other organisms
☒	☐	Human research participants
☒	☐	Clinical data

Methods

n/a	Involved in the study	
☒	☐	ChIP-seq
☒	☐	Flow cytometry
☒	☐	MRI-based neuroimaging

Animals and other organisms

Policy information about studies involving animals; ARRIVE guidelines recommended for reporting animal research

Laboratory animals	We used three male Long-Evans rats (ranged from 2 to 8 months).
Wild animals	This study did not involve wild animals.
Field-collected samples	This study did not involve samples collected from the field.
Ethics oversight	All protocols were approved by the Institutional Animal Care and Use Committees of Rutgers University.

Note that full information on the approval of the study protocol must also be provided in the manuscript.
