# Supplementary material for: Reconstructing neuronal circuitry from parallel spike trains
Source: Nat Commun. 2019 Oct 2;10:4468. doi: 10.1038/s41467-019-12225-2 (PMC6775109; doi:10.1038/s41467-019-12225-2)
Supplement: Supplementary file 4 — Source Data [file 41467_2019_12225_MOESM4_ESM.zip › js/index.html]

Reconstructing neuronal circuitry from spike trains


##### Reconstructing neuronal circuitry from spike trains.

Web-application developed by Masahiro Naito, Ryota Kobayashi, and Shigeru Shinomoto (Kyoto University)

This application program builds a neuronal circuit diagram from parallel spike trains. By uploading recorded spike data, you can obtain a connection matrix, whose elements are interneuronal connections estimated in units of postsynaptic potentials. This estimation is based on the generalized linear model applied to cross correlation (GLMCC) [Reference in the below]. Python code is also available.

Sample1

Sample2

Sample3

Sample4

Upload your data

How to prepare your data

Prepare your data in a {.txt} format. The data consists of a set of neuronal spike trains separated by semicolons {;} as

```
{spike train of the 1st neuron}; 
{spike train of the 2nd neuron};
...
{spike train of the Nth neuron};
```

Each {spike train} is given as a series of spike times separated by a newline, a comma, or a space. A sample in which spike times are separated by a newline (and spike trains are separated by a semicolon) is shown below

```
1692.529986
2372.809986
2682.789986
...
;
1396.319986
1405.629986
1713.209986
...
;
```

In our default setting, spike times should be represented in a unit of [msec], but you can change the setting into [sec] or [µsec]. A sample data may be downloaded from here.

Change default settings and model parameters

γ

τ

###### significance level α

0.01
0.001
original

###### other parameters

Experimental data

bins

WIN

ds

start

end

min\_s

###### unit of time

sec
msec
µsec

Estimate connectivity

0%

log  

---


100%
125%
150%
200%
300%
500%

csv

table.svg

circuit.svg

E-I dominance

##### cross-correlogram:

detected cross-correlograms.svg

---


This application program was developed by Masahiro Naito. Python code was developed by Junichi Haruna. An original program was formulated by Ryota Kobayashi. The analysis was directed by Shigeru Shinomoto.

[Reference] R. Kobayashi, S. Kurita, K. Kitano, K. Mizuseki, B. J. Richmond, and S. Shinomoto, Reconstructing neuronal circuitry from parallel spike trains. The preprint is available from bioRxiv, 334078.

Version 2.1: 2019/04/30. Visitors since 2018/11/29:
